# Host immunological responses facilitate development of SARS-CoV-2 mutations in patients receiving monoclonal antibody treatments

**DOI:** 10.1172/JCI166032

**Published:** 2023-03-15

**Authors:** Akshita Gupta, Angelina Konnova, Mathias Smet, Matilda Berkell, Alessia Savoldi, Matteo Morra, Vincent Van averbeke, Fien H.R. De Winter, Denise Peserico, Elisa Danese, An Hotterbeekx, Elda Righi, mAb ORCHESTRA working group, Pasquale De Nardo, Evelina Tacconelli, Surbhi Malhotra-Kumar, Samir Kumar-Singh

**Affiliations:** 1 Molecular Pathology Group, Cell Biology & Histology, Faculty of Medicine and Health Sciences and; 2 Laboratory of Medical Microbiology, Vaccine & Infectious Disease Institute, University of Antwerp, Antwerp, Belgium.; 3 Division of Infectious Diseases, Department of Diagnostics and Public Health and; 4 Section of Clinical Biochemistry, University of Verona, Verona, Italy.; 5 The mAb ORCHESTRA working group is detailed in Supplemental Acknowledgments.

**Keywords:** COVID-19, Cellular immune response

## Abstract

**Background:**

The role of host immunity in emergence of evasive SARS-CoV-2 Spike mutations under therapeutic monoclonal antibody (mAb) pressure remains to be explored.

**Methods:**

In a prospective, observational, monocentric ORCHESTRA cohort study, conducted between March 2021 and November 2022, mild-to-moderately ill COVID-19 patients (*n* = 204) receiving bamlanivimab, bamlanivimab/etesevimab, casirivimab/imdevimab, or sotrovimab were longitudinally studied over 28 days for viral loads, de novo Spike mutations, mAb kinetics, seroneutralization against infecting variants of concern, and T cell immunity. Additionally, a machine learning–based circulating immune-related biomarker (CIB) profile predictive of evasive Spike mutations was constructed and confirmed in an independent data set (*n* = 19) that included patients receiving sotrovimab or tixagevimab/cilgavimab.

**Results:**

Patients treated with various mAbs developed evasive Spike mutations with remarkable speed and high specificity to the targeted mAb-binding sites. Immunocompromised patients receiving mAb therapy not only continued to display significantly higher viral loads, but also showed higher likelihood of developing de novo Spike mutations. Development of escape mutants also strongly correlated with neutralizing capacity of the therapeutic mAbs and T cell immunity, suggesting immune pressure as an important driver of escape mutations. Lastly, we showed that an antiinflammatory and healing-promoting host milieu facilitates Spike mutations, where 4 CIBs identified patients at high risk of developing escape mutations against therapeutic mAbs with high accuracy.

**Conclusions:**

Our data demonstrate that host-driven immune and nonimmune responses are essential for development of mutant SARS-CoV-2. These data also support point-of-care decision making in reducing the risk of mAb treatment failure and improving mitigation strategies for possible dissemination of escape SARS-CoV-2 mutants.

**Funding:**

The ORCHESTRA project/European Union’s Horizon 2020 research and innovation program.

## Graphical abstract

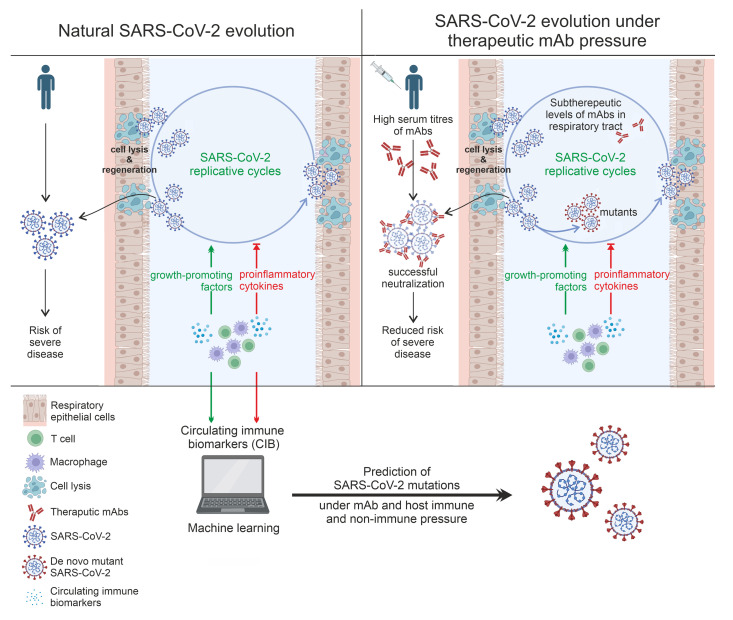

## Introduction

The coronavirus replication machinery encodes proofreading functions that result in fewer errors compared with other RNA viruses; however, multiple SARS-CoV-2 variants of concern (VOCs) have emerged throughout the pandemic carrying VOC-defining mutations. For example, Alpha (B.1.1.7), Beta (B.1.351), Gamma (P.1), Delta, Zeta, Eta, Theta, Iota, and Omicron variants have been shown to carry distinct sets of mutations that evade existing natural neutralizing antibody responses (1–4).

SARS-CoV-2 mutation rates are higher in immunocompromised or severely ill patients who show prolonged SARS-CoV-2 infections or carriage (5–12). Immunocompromised individuals are also unable to develop sufficient antibody titers after administration of COVID-19 vaccines. To tackle this, synthetic neutralizing monoclonal antibodies (mAbs) against SARS-CoV-2 targeting the Spike (S) protein have been developed that demonstrate clinical benefit for mild-to-moderately ill COVID-19 patients at high risk of developing severe disease (13–20). For example, the first widely available mAb, bamlanivimab, which targets an epitope on the receptor-binding domain (RBD), led to a reduced rate of hospitalization, ICU admission, and mortality compared with usual care (21). The addition of etesevimab to bamlanivimab resulted in improved clinical outcomes due to overlapping binding epitopes within the RBD of the S protein, concomitant with the emergence of SARS-CoV-2 VOCs, mainly B.1.351 and P.1 (22). The success of combination mAb therapy and decreasing efficacies against emerging variants led to use of casirivimab/imdevimab, with distinct binding sites in S RBD, in at-risk populations, resulting in decreased rates of hospitalization (23). As the pandemic evolved and new VOCs were identified, sotrovimab was developed with a modified Fc domain along with an increased half-life (13, 14). Recently, an intramuscularly administered combination of noncompeting antibodies tixagevimab and cilgavimab, again with distinct binding sites, has also been introduced in patient care (16). These modifications target highly conserved S epitopes, causing conformational transitions necessary for association with the receptor angiotensin-converting enzyme 2 (ACE2) (15, 16), resulting in reduced risk of disease progression and death (13, 24).

Several reports have also identified de novo mutations under therapeutic mAb pressure, including E484Q/K and Q493K/R under bamlanivimab/etesevimab pressure (25–27) and P337R/S, E340D/K/V, and G446S/V under casirivimab/imdevimab and/or sotrovimab pressure (28–31). However, despite the widespread use of mAbs, these studies are rather few and were conducted in limited patient numbers. Moreover, to our knowledge the role of host immune pressure in selection of mAb-driven de novo SARS-CoV-2 S RBD mutations has not been explored so far.

Here, we characterize the development of SARS-CoV-2 S RBD mutations in patients treated with bamlanivimab, bamlanivimab/etesevimab, casirivimab/imdevimab, or sotrovimab in relation to their neutralization potential against SARS-CoV-2 VOCs. We focus on natural humoral and cellular host immunity, including responses mediated by cytokines and other correlates of adaptive evolution.

## Results

### Immunocompromised COVID-19 patients receiving early mAb therapy continue to display significantly higher viral loads compared with nonimmunocompromised patients.

The H2020-funded ORCHESTRA project (Connecting European Cohorts to Increase Common and Effective Response to SARS-CoV-2 Pandemic) includes work package 2 (WP2), prospectively enrolling high-risk patients receiving early treatment for symptomatic COVID‑19. Clinical efficacies of bamlanivimab, bamlanivimab/etesevimab, casirivimab/imdevimab, or sotrovimab in 740 mild-to-moderate nonhospitalized COVID-19 patients have been described (19, 20) (for eligibility criteria, see Supplemental Table 1; supplemental material available online with this article; https://doi.org/10.1172/JCI166032DS1). From this WP2 cohort, patients were prospectively invited to a substudy assessing immunological and virological responses to mAbs studied by WP6 of the ORCHESTRA project.

Overall, 204 patients receiving bamlanivimab (*n* = 45), bamlanivimab/etesevimab (*n* = 108), casirivimab/imdevimab (*n* = 17), or sotrovimab (*n* = 34) were enrolled (Table 1). Patients were assessed and sampled before mAb infusion (day 0, D0) and after treatment on D2, D7, and in 98 patients on D28. The maximum study length of 28 days was chosen as the mean duration of SARS-CoV-2 RNA shedding from the upper respiratory tract and has been estimated as not more than 17 days (32, 33). Patient groups did not differ significantly in WHO progressive severity score (34). The median age of the total study cohort was 64 years (interquartile range [IQR]: 62–74) and 53.9% of the enrolled patients were males. During the 28-day follow-up, 28 patients (28 of 204; 13.7%) were hospitalized for severe COVID-19 (bamlanivimab: 8 of 45 [17.7%]; bamlanivimab/etesevimab: 20 of 108 [18.5%]) and 3 of 204 patients died (1.5%). For patient characteristics, see Table 1.

SARS-CoV-2 whole-genome sequencing revealed variants belonging to 5 distinct clades, of which the most frequent were 20I/Alpha (*n* = 161), 21K/Omicron (*n* = 27), and 21L/Omicron (*n* = 7). Patients receiving bamlanivimab, bamlanivimab/etesevimab, or casirivimab/imdevimab mostly carried Alpha subvariants (B.1.1.7, 146 of 170; Q.4, 15 of 170) at baseline except for 3 patients who carried 20A/B.1.462 or 20D/C.36.3 (Table 1). All patients treated with sotrovimab carried Omicron subvariants, the most common being 21K/BA.1 with the S:R346K substitution (*n* = 14; BA.1.1, BA.1.1.1), followed by 21K/BA.1 (*n* = 13; BA.1, BA.1.17, BA.1.17.2) and 21L/BA.2 (*n* = 7; BA.2, BA.2.9).

**Figure 1:**
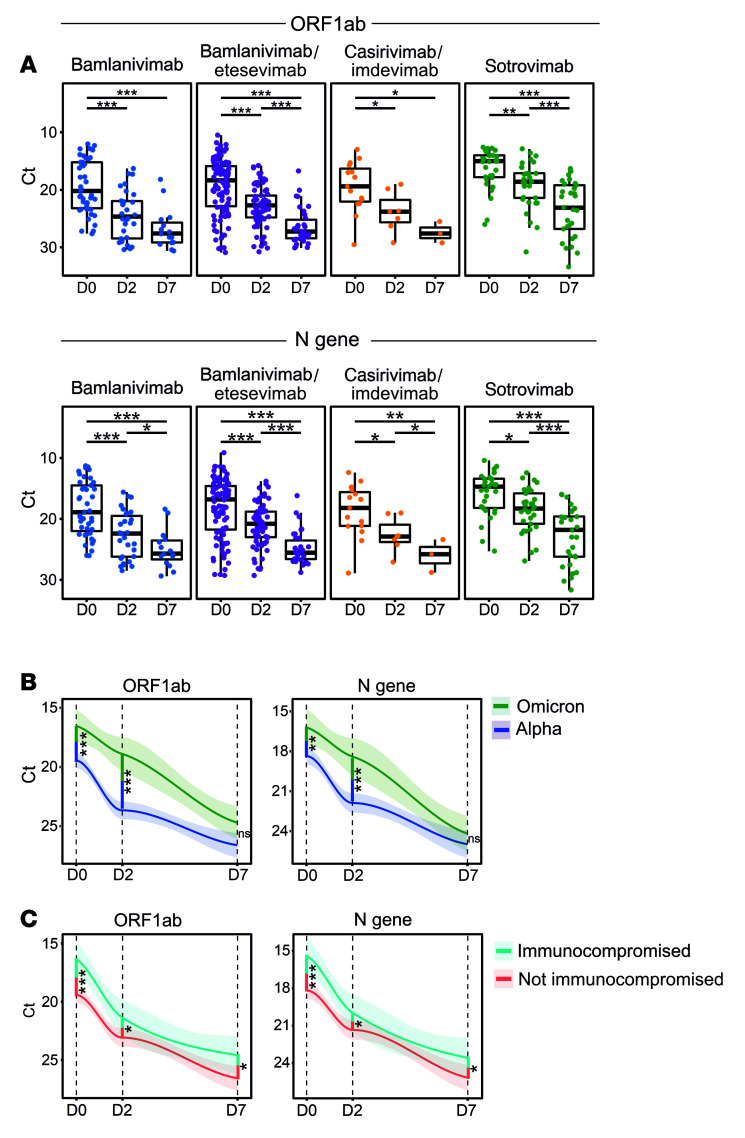
Immunocompromised and Omicron-infected COVID-19 patients display higher viral loads after mAb administration. RT-qPCR detection of SARS-CoV-2 was performed on nasopharyngeal swab samples collected on D0, D2, and D7 from patients treated with different therapeutic mAbs. (**A**) A steady increase in Ct values was observed over 7 days for all mAb-treated groups. Box-and-whisker plots indicate median (middle line), 25th and 75th percentiles (box boundary), and 5th and 95th percentiles (whiskers). All data points, including outliers, are displayed. (**B**) Overall, patients carrying Omicron (BA.1, BA1+R346K, or BA.2) displayed higher viral loads than patients carrying Alpha subvariants (B.1.1.7 or Q4). (**C**) Immunocompromised patients carried higher viral loads, irrespective of the infecting SARS-CoV-2 variant and mAb treatment. Line graphs in **B** and **C** represent smoothed conditional means, with shaded areas displaying 95% CIs for all measured time points. Cross-sectional and longitudinal statistical comparisons were performed using Mann-Whitney followed by Bonferroni’s post hoc correction. *P < 0.05; **P < 0.01; ***P < 0.001. NS, nonsignificant; D0, sample collected prior to mAb infusion; D2, 2 ± 1 days after mAb infusion; D7, 7 ± 2 days after mAb infusion. A limited number of nasopharyngeal swab samples were collected on D28 (*n* = 9) across all 4 mAb therapy groups and were therefore excluded from this analysis. See Supplemental Tables 2 and 3 for details on Ct values at each time point.

Differences in viral loads in patients undergoing different mAb treatments were longitudinally studied by comparing cyclic threshold (Ct) values for open reading frame 1 ab– (ORF1ab-), nucleocapsid (N) protein–, and S protein–encoding genes by quantitative real-time reverse transcription PCR (RT-qPCR). A gradual, significant increase in Ct values was observed for all gene targets, indicating a decreasing viral load (Figure 1A and Supplemental Tables 2 and 3). Due to the S:Δ69/70 deletion in Alpha (B.1.1.7, Q.4) and BA.1 (+R346K)/Omicron subvariants, most samples were qPCR negative for the *S* gene. Compared with patients infected with Alpha subvariants, patients carrying Omicron subvariants showed significantly higher viral loads before mAb infusion (D0) that stayed significantly higher until 48 hours after mAb infusion (D2 time point; Figure 1B).

As several studies have shown that immunocompromised individuals show a prolonged carriage of SARS-CoV-2 (5, 7), we investigated whether these patients receiving mAb therapy also carried higher viral loads. Immunocompromised status was defined clinically on the basis of patients on active immunosuppressive treatment for cancer, organ transplants, and/or immunological diseases, as described previously (19, 20) (Table 1 and Supplemental Table 1). We show that immunocompromised patients had higher viral loads at the time of enrollment irrespective of the mAb treatment (ΔCt 3.03 and 2.76 for *ORF1ab* and *N*, respectively; *P* ≤ 0.001). Remarkably, significantly higher viral loads persisted in immunocompromised patients at both D2 and D7 time points (ΔCt on D7, 1.89 and 1.79 for *ORF1ab* and *N*, respectively; *P* ≤ 0.03) (Figure 1C). These data suggest that prolonged viral shedding occurs in immunocompromised COVID-19 patients with mild-to-moderate disease despite receiving mAb therapies.

### Immunocompromised patients display higher rates of SARS-CoV-2 S RBD mutations.

To study the emergence of amino acid–substituting SARS-CoV-2 mutants in response to mAb treatment, 204 patients were studied longitudinally for mutations occurring on D2 or D7, compared with the pretherapy (D0) time point. Overall, 35 patients (17.2%) developed nonsynonymous mutations at 43 unique positions in the SARS-CoV-2 genome, which resulted in 48 unique amino acid substitutions. Seventeen patients developed mutations across 26 unique positions randomly distributed across the SARS-CoV-2 genome (ORFs 1ab, 3a, and 7ab, or the *M* and *N* genes), and each position was only found to be mutated in 1 patient each (Supplemental Figure 1 and Supplemental Table 4). The remaining 22 of 48 nonsynonymous mutations occurred within the *S* gene in 22 patients overall. In total, 16 unique amino acid substitutions occurred in S RBD (residues 319–541) in a total of 17 patients. All mutations identified in patients receiving bamlanivimab with or without etesevimab have been previously reported, whereas most emerging S RBD mutants in the sotrovimab-treated group were novel to the best of our knowledge and occurred in clusters (see below). As RT‑qPCR errors have been suggested to be amplified to high allele frequencies resulting in sequencing errors, especially under low viral load conditions (8, 11), all nonsynonymous S RBD mutations in sotrovimab-treated patients were reconfirmed by either Sanger or repeated NextSeq sequencing on independently extracted RNA.

**Figure 2:**
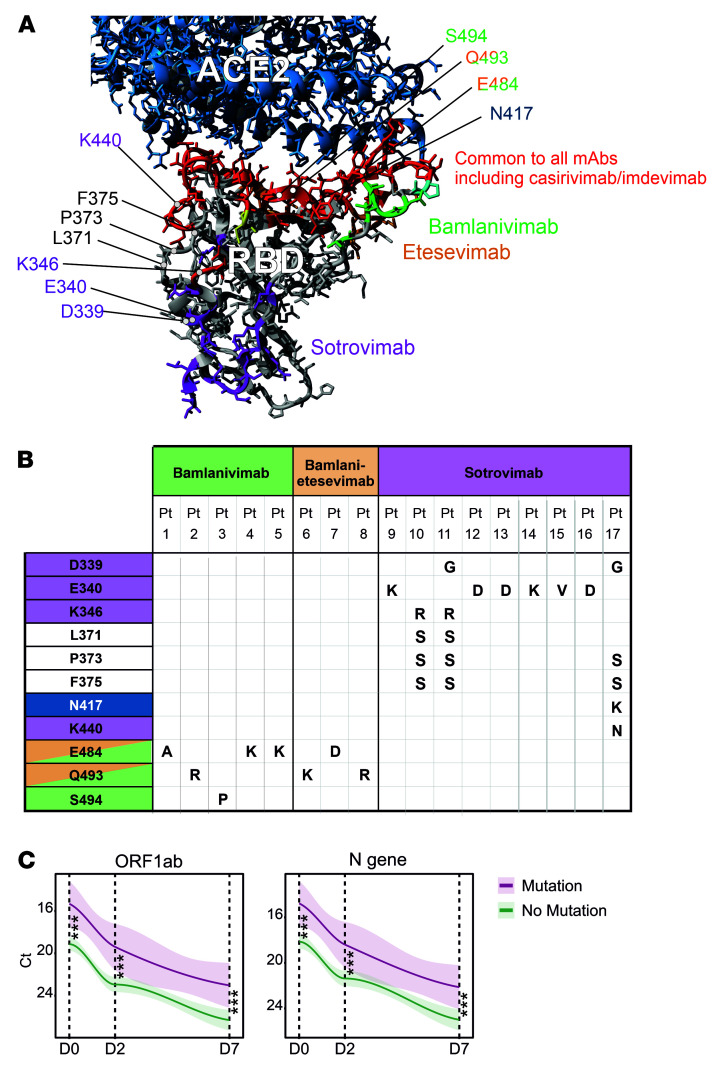
De novo SARS-CoV-2 S RBD mutations evolving under mAb pressure. (**A**) Schematic quaternary structure of the SARS-CoV-2 S RBD protein when bound to the human (h)ACE2 receptor (PDB: 6M0J). Key RBD-binding sites of bamlanivimab, etesevimab, and sotrovimab are highlighted in the protein structure with corresponding colors. Binding sites common to all mAbs, including casirivimab and imdevimab, are indicated in red, whereas hACE2 is highlighted in blue. (**B**) SARS-CoV-2 genomes longitudinally isolated from patients receiving mAb therapy were screened for the emergence of de novo mutations resulting in amino acid substitutions in the S RBD region. Most commonly, escape mutants occurred in residues harbored within the respective mAb binding site. Pt, patient. (**C**) Patients developing S RBD mutations were found to harbor significantly higher viral loads at all time points. Cross-sectional statistical comparisons were performed using the Mann-Whitney test. Lines represent smoothed conditional means and shaded areas display 95% CIs for all measured time points. ***P < 0.005. For more details on nonsynonymous de novo changes and sample numbers, see Supplemental Figures 1 and 7 and Supplemental Table 4.

A remarkable mutational homogeneity was identified wherein the same mutations developed independently in SARS-CoV-2 S RBD in different patients under mAb pressure. For instance, all 8 patients developing S RBD mutations receiving bamlanivimab or bamlanivimab/etesevimab involved only 3 residues (E484, Q493, and S494; Figure 2, A and B, and Supplemental Figure 2). Among these, Q493R/K was present in 3 patients and involved a residue common to both bamlanivimab and etesevimab binding sites, suggesting a potential loss of function of binding of both mAbs to the mutated SARS-CoV-2 S protein. Similarly, mutations identified in sotrovimab-treated patients were present in either ACE2 (N417) or sotrovimab binding sites (D339, E340, R346, and K440), except for 3 mutations involving residues L371, P373, and F375 identified in 3 patients (Figure 2B). These mutations involved alternate residues of SARS-CoV-2 S RBD and were notably substituted to serine, consistent with the Wuhan-Hu-1 protein sequence. Two additional reversions (D339G and K346R) were identified in the sotrovimab-treated group, the latter mutation reversing the BA.1.1‑defining R346K substitution (35).

Notably, a highly diverse *S* gene mutation rate was also observed under the different mAb treatment/variant combinations. For example, 9 of 34 (26.5%) patients carrying Omicron and receiving sotrovimab developed S RBD mutations, which was significantly higher compared with patients receiving other mAb treatments and carrying Alpha or other variants, i.e., 5 of 45 (11.1%) patients receiving bamlanivimab, 3 of 108 (2.8%) receiving bamlanivimab/etesevimab, and none (0 of 17) in the casirivimab/imdevimab group (Pearson’s χ^2^ = 21.005; *n* = 204; degrees of freedom [df] = 3; *P* < 0.001).

Interestingly, patients with de novo S RBD mutations had approximately 10-fold increased burden of viral genetic material on D0 compared with patients without SARS-CoV-2 mutations across all mAb treatment groups (average ΔCt for *ORF1ab* and *N* = 3.37, range 2.9–3.8, *P* ≤ 0.001), and remained elevated at both D2 and D7 time points (*P* < 0.005 for both time points; Figure 2C). These data suggest that higher viral loads predispose to development of SARS-CoV-2 mutations. As immunocompromised individuals carried higher viral loads, we further assessed whether these individuals are more likely to develop S RBD mutations. Out of 17 patients who developed SARS-CoV-2 S RBD mutations, 6 were immunocompromised (35.3%), while only 11 patients of 170 nonimmunocompromised patients developed mutations (6.5%). Using χ^2^ and odds ratio (OR) as a test and measure of association, respectively, we showed that immunocompromised individuals treated with mAbs had significant 3-fold greater odds of developing S RBD mutations compared with nonimmunocompromised patients (Pearson’s χ^2^ = 4.633, *n* = 204, df = 1, *P* = 0.031, OR = 3.097, 95% CI [1.060, 9.050]). Together, these data suggest that COVID-19 patients receiving mAb therapy develop S RBD mutations that are not only mAb therapy or variant dependent, but the rate of intrahost S mutations is also substantially increased in patients who are immunocompromised.

### Therapeutic mAb titers are not directly associated with development of S RBD mutations.

**Figure 3:**
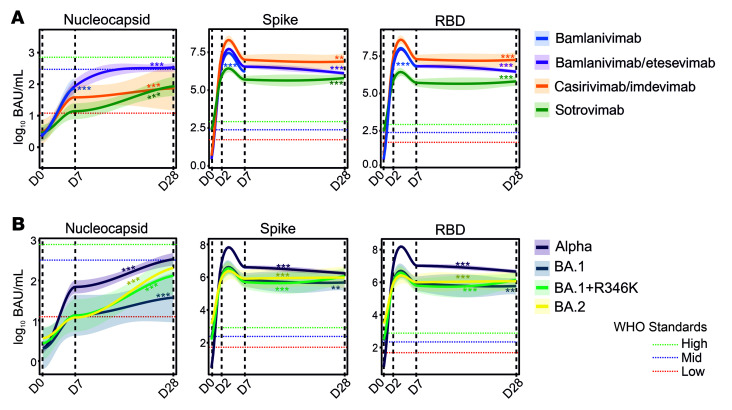
Temporal evolution of anti-N, anti-S, and anti-RBD serology titers in patients receiving mAb therapies. (**A**) Natural immunity was assessed based on anti-N titers, revealing a gradual increase through D28. High anti-S and anti-RBD titers due to therapeutic mAb administration persisted from D2 to D28 in patients in all treatment groups. (**B**) Similarly, high anti-S and anti-RBD titers were observed in patients carrying Omicron subvariants (BA.1, BA1+R346K, or BA.2) receiving sotrovimab monotherapy. Red, green, and blue dotted lines indicate SARS-CoV-2 WHO reference standard values for low, medium, and high antibody titers, respectively. Line graphs in **A** and **B** represent conditional means and shaded areas displaying 95% CIs for all measured time points. Linear mixed models were utilized to investigate evolution of antibody titers over time for different mAbs, with asterisks indicating significance of the slopes of the curves. **P < 0.01, ***P < 0.001. For more details on serology in patients with or without vaccination and sample numbers, see Supplemental Table 5 and Supplemental Figure 7.

We investigated anti-S and anti-RBD titers for different mAb treatment groups along with naturally developing anti-N titers at all time points. As sotrovimab was given to patients who were vaccinated (73.5%; 14 days after dose, ≥2 doses, *n* = 25; see Table 1), we first showed that, as expected, vaccine-related anti-S and anti-RBD titers, but not anti-N titers, were significantly elevated in the sotrovimab group at time of enrollment (D0) (Supplemental Figure 3). To address whether intervention with mAbs targeting SARS-CoV-2 could dampen the development of natural immunity, we studied anti-N titers that are not expected to be affected by therapeutic mAbs. A significant rise in anti-N titers was observed for all treatment groups, although the increase from preinfusion titers (D0) to titers on D7 and D28 was smaller for the casirivimab/imdevimab and sotrovimab therapy groups compared with all others (Figure 3A and Supplemental Table 5). No significant difference in anti-S and anti-RBD titers was identified between patients infected with dominant circulating SARS-CoV-2 variants, including Omicron subvariants (Figure 3B).

To study whether therapeutic antibodies could be linked to development of SARS-CoV-2 S RBD mutations, we first showed that prior to therapy (D0), anti-S or anti-RBD titers were not significantly different in S RBD mutation carriers (*n* = 204; anti-S, *F* = 0.032, *P* = 0.859; anti-RBD, *F* = 0.140, *P* = 0.708). Similarly, we studied whether levels of therapeutic mAbs in blood could be associated with S RBD mutations in our cohort. At the first posttherapy time point (D2), the average titers for anti-RBD and anti-S were 11.5 and 6.4 million binding antibody units (BAU)/mL, respectively. By comparison, the WHO International SARS-CoV-2 Antibody Standards for “High blood immunoglobin” corresponds to the anti-RBD titer of 817 BAU/mL and anti-S titers of 832 BAU/mL. Both anti-S and anti-RBD titers dropped on D7 and further on D28 for the majority of the mAb treatment groups, but average anti-RBD and anti‑S titers on D28 remained at 5.8 and 2.9 million BAU/mL, respectively. The bioavailability of IgGs at the mucosal barrier, where the mAb-selection pressure likely exists, is not known; however, with more than 10,000 greater “free” therapeutic mAb titers measured in blood than those required for effective virus neutralization, expectedly, we did not find any direct selective pressure of therapeutic mAbs in the development of SARS-CoV-2 S RBD mutations.

### Neutralizing capacities of mAbs are (co)drivers of development of escape mutants.

**Figure 4:**
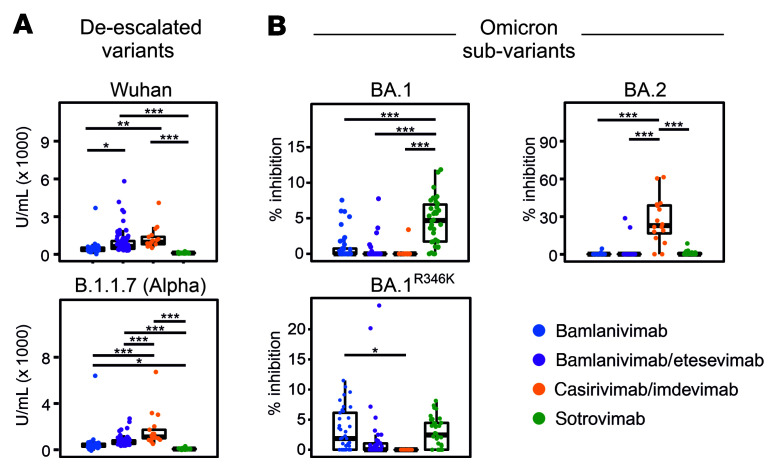
Anti-S neutralization capacity of bamlanivimab, bamlanivimab/etesevimab, casirivimab/imdevimab, and sotrovimab. Neutralization capacity was measured against (**A**) deescalated variants and (**B**) Omicron subvariants on D2. Sotrovimab monotherapy proved most effective in neutralizing BA.1. Bamlanivimab showed increased neutralizing activity against BA.1. Casirivimab/imdevimab combination therapy proved highly effective in neutralization of BA.2. Box-and-whisker plots indicate median (middle line), 25th and 75th percentiles (box boundary), and 5th and 95th percentiles (whiskers). All data points, including outliers, are displayed. Statistical assessments were performed using pairwise 2-tailed *t* tests with Bonferroni’s post hoc correction. *P < 0.05; **P < 0.01; ***P < 0.001. For details on tested variants of concern and sample numbers, see Supplemental Table 6 and Supplemental Figure 7.

We further investigated whether development of S RBD mutations is linked to the neutralization potential of different mAbs. Studying neutralizing capacities of the 4 mAb regimens in an ACE2 neutralization assay, we first showed a highly significant difference by which these mAbs neutralize 5 past or currently circulating SARS-CoV-2 variants (Figure 4 and Supplemental Figure 4). Casirivimab/imdevimab appeared to have the highest neutralizing activity against most variants, including Wuhan-Hu-1, Alpha, and Omicron/BA.2 variants. Sotrovimab monotherapy showed best neutralization results against Omicron BA.1 (including BA.1+R346K sublineages); however, neutralizing activity of sotrovimab against BA.2 was lower compared with BA.1 and BA.1+R346K (*P* < 0.05), as shown previously where sotrovimab retained activity against both BA.1 and BA.1+R346K, but its activity against BA.2 dropped 27-fold (35).

Remarkably, in the sotrovimab-treated group, both BA.1 and BA.2 infections were observed, allowing us to assess whether the neutralizing potential of mAbs could increase the likelihood of development of S RBD mutations. We show that for BA.1 and BA.1+R346K groups against which sotrovimab shows good neutralizing capacity, 9 of 27 (33.3%) of patients developed mutations. On the other hand, none of the patients in the BA.2 group (0 of 7) developed S RBD mutations against which sotrovimab shows poor neutralization capacity; these data were also statistically significant (likelihood ratio = 4.97, *n* = 34; df = 1, *P* = 0.026). Importantly, a higher proportion of immunocompromised patients (4 of 7, 57.1%) were present in the BA.2 group that did not develop mutations compared with the BA.1 group (13 of 27, 48.1%) (Spearman’s correlation, covariance = 0.201, *P* = 0.708). These data strongly suggest that seroneutralization capacities of therapeutic mAbs are independently linked with development of SARS-CoV-2 escape mutants.

### Natural adaptive T cell immunity is associated with development of SARS-CoV-2 escape mutants.

Existing immunity against SARS-CoV-2 infections as a result of current or past exposure, vaccination against SARS-CoV-2, or human immune system variations could strongly influence the disease outcome in patients receiving different mAb regimens. To address the impact of mAb therapies on T helper (Th) cell immunity, lymphocytes collected on D0 and D28 were stimulated by either a SARS-CoV-2 N or a complete S protein peptide pool (see Supplemental Methods). CD4^+^ Th cell activation was subsequently studied by both a general marker, CD154 (CD40L), and by IFN-γ, a cytokine marker of antigen-reactive Th cells.

**Figure 5:**
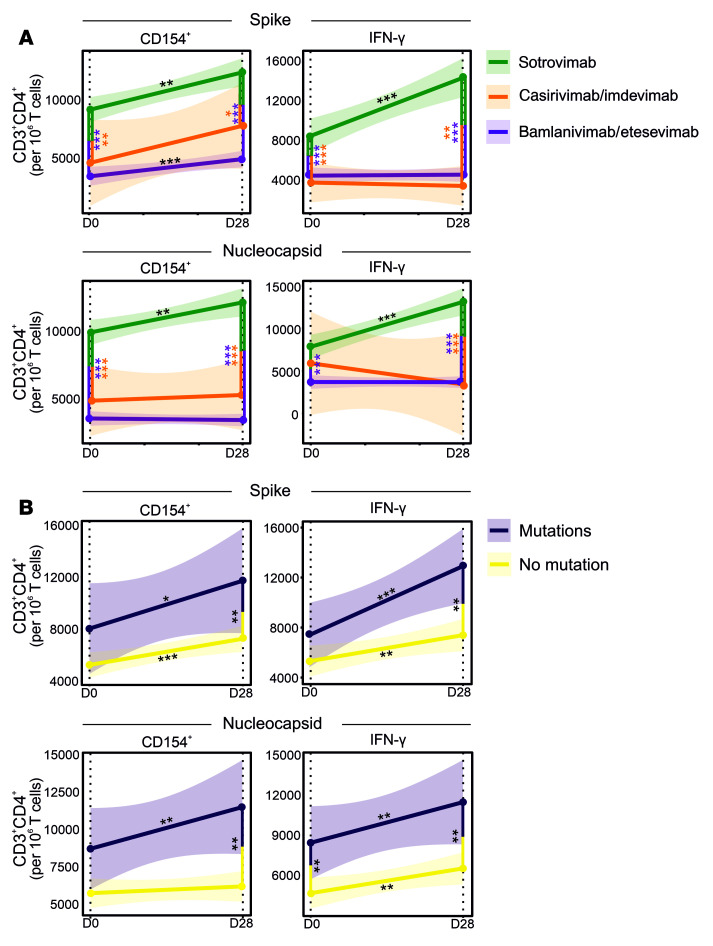
Longitudinal T cell responses in patients receiving mAb therapy. Evolution of IFN-γ and CD154 expression in SARS-CoV-2 S– and Nucleocapsid–stimulated CD4^+^ T cells in patients was studied over 28 days after receiving bamlanivimab/etesevimab, casirivimab/imdevimab, or sotrovimab. (**A**) Patients receiving sotrovimab therapy show a consistent significant increase in T cell expression during the first 28 days after mAb administration. For the utilized gating strategy, refer to Supplemental Figure 8. (**B**) Patients with de novo mutations in the SARS-CoV-2 S RBD region show an increased T cell expression compared with those without. Linear mixed models were utilized to investigate evolution of Th cell immunity over time between the different mAb groups. Regression curves represent smoothed conditional means and shaded areas display 95% CIs for all measured time points, with asterisks on lines representing the significance of the slopes. Vertical lines with asterisks represent the significance of pairwise comparisons between patients with or without de novo mutations before mAb treatment (D0) and after 28 days of treatment (D28). *P < 0.05; **P < 0.01; ***P < 0.001.

On D0, the number of both S- and N-activated Th cells was significantly higher in the sotrovimab-treated group (*n* = 25) compared with bamlanivimab/etesevimab (*n* = 42) and casirivimab/imdevimab (*n* = 5) groups (*P* < 0.01; Figure 5A). While the higher number of S-activated Th cells in sotrovimab-treated patients could be explained by vaccination, with most of the patients in this group being fully vaccinated, a concurrently higher number of N-activated Th cells in sotrovimab-treated patients suggests a likely higher rate of prior SARS-CoV-2 exposure, as vaccination was administered to patients in this group later in the pandemic. Furthermore, over 28 days, the sotrovimab-treated group also showed a significantly higher increase in N-activated CD4^+^IFN-γ^+^ cells compared with bamlanivimab/etesevimab and casirivimab/imdevimab groups (*P* < 0.001). These data suggest that sotrovimab does not substantially curb development of natural immunity and fits well with the higher viral clearance observed in the sotrovimab-treated group carrying Omicron subvariants compared with bamlanivimab/etesevimab and casirivimab/imdevimab groups carrying Alpha subvariants (Figure 5A and Figure 1B).

Addressing whether mAb-induced S RBD mutations were associated with Th cell immunity, we further showed that patients developing mutations had slightly higher proportions of N-activated CD4^+^CD154^+^ and CD4^+^IFN-γ^+^ cells before mAb treatment, which was statistically significant for CD4^+^IFN-γ^+^ cells (*P* < 0.05; Figure 5B). However, strikingly, patients exhibiting de novo mutations also developed stronger Th cell immunity over 28 days, with significantly increased S- and N-activated CD4^+^CD154^+^ and CD4^+^IFN-γ^+^ cells on D28 (*P* < 0.01). Although whether activated CD4^+^ Th cells could stimulate naive B cells to produce specific antibodies against the mutant virus, or whether preexisting high-affinity antibodies induced by previous vaccinations in sotrovimab-treated patients bias memory B cell selection in contributing to the increased frequency of SARS-CoV-2 mutants (36, 37) is not known, our data strongly support the premise that the identified de novo mutations in the SARS-CoV-2 S protein are indeed escape mutations that evade therapeutic mAb neutralization, thereby facilitating a more natural progression of disease and resulting in more robust SARS-CoV-2–specific Th cell immunity.

### Host immune profile as a predictor of S RBD escape mutants.

Studies have shown that proinflammatory cytokines, when uncontrolled and exaggerated, can lead to immunopathogenesis such as cytokine release syndrome disorder; however, under homeostatic conditions they are believed to play a major role in the control and resolution of SARS-CoV-2 infection (38, 39). Moreover, cytokines along with growth factors are critical to fundamental homeostatic processes such as wound healing and tissue repair (40). We hypothesized that a host environment that is (a) less hostile to the virus and (b) facilitates tissue repair would together allow boosted cell infection cycles for rapid viral evolution under mAb pressure. To address this hypothesis, we studied 40 blood cytokines, chemokines, and growth factors as part of circulating immune-related biomarkers (CIBs) involved in either COVID-19 pathogenesis and/or wound healing.

**Figure 6:**
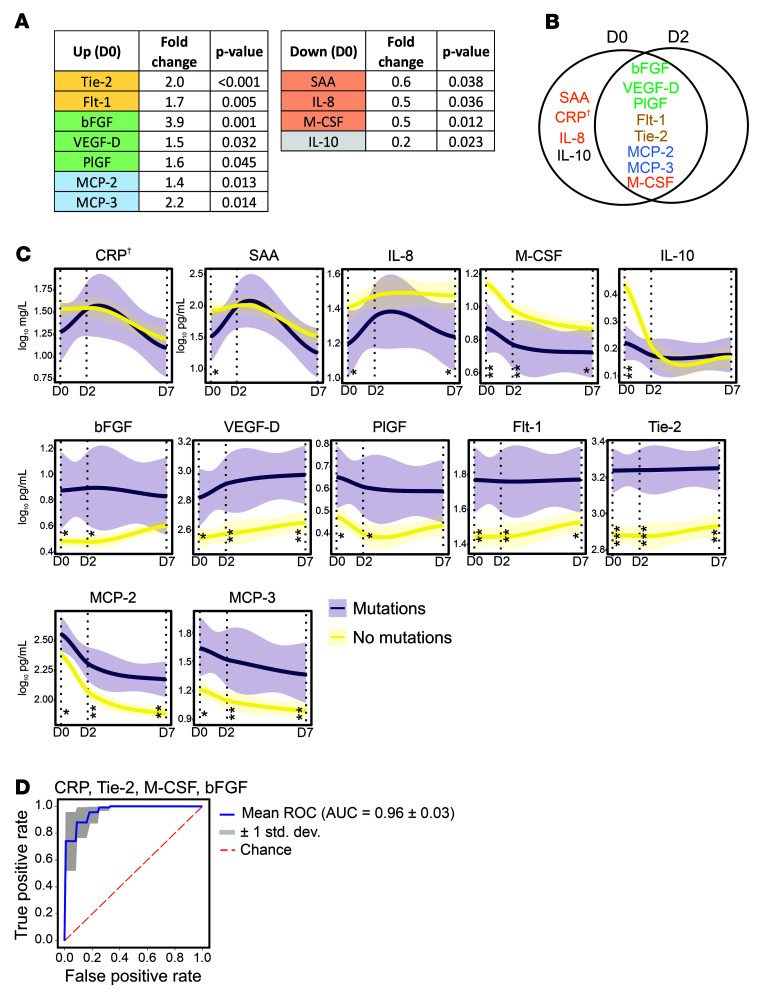
Circulating immune-related biomarkers (CIBs) in COVID-19 patients receiving mAb therapy. (**A**) Several CIBs were significantly up- or downregulated on D0 in COVID-19 patients who developed SARS-CoV-2 S RBD mutations after administration of mAb treatments, compared with those who did not. (**B**) Eleven CIBs were significantly altered on D0 in patients with de novo S RBD mutations, for which the majority (*n* = 8) were also altered on D2. (**C**) Temporal evolution of CIBs altered in patients, with or without de novo mutations, receiving mAb therapy through day 7 after treatment. Lines represent smoothed conditional means and shaded areas display 95% CIs for all measured time points. *P* values refer to significance of the slope of the regression lines. Vertical lines with asterisks represent the significant difference between CIB levels at the specified time points. (**D**) Receiving operator characteristic (ROC) curve in a random forest classifier model with synthetic minority oversampling technique (SMOTE) for the prediction of mutation versus no-mutation are depicted for D0. *P < 0.05, **P < 0.01, ***P < 0.001. †Not significant. For details on the progression of CIBs from D0 to D7 and sample numbers, see Supplemental Figures 5 and 7.

Significant changes between different treatment groups occurred in the levels of 34 of 40 (85.0%) cytokines (Supplemental Figure 5) that are also linked to infection with different SARS-CoV-2 variants. We further utilized area under the curve receiver operating characteristic (AUROC) analysis to discriminate between patients developing de novo S RBD mutations from those who did not or those who rapidly cleared the virus. AUROC for CIBs just before mAb administration identified 11 biomarkers to be significantly altered. Among these, 8 biomarkers were significantly increased in patients developing mutations on D2, and included angiogenic growth factors (basic fibroblast growth factor [bFGF], placental growth factor [PlGF], and vascular endothelial growth factor D [VEGF-D]), angiogenic factors’ receptors (angiopoietin receptor 1 [Tie-2] and vascular endothelial growth factor receptor 1 [Flt-1]), and drivers of healing responses through macrophages (monocyte chemoattractant protein 2 [MCP-2] and MCP-3) (41) (Figure 6A). The 4 biomarkers that were significantly downregulated were acute-phase inflammatory marker serum amyloid A (SAA), neutrophil chemokine IL-8, immunomodulatory marker IL-10, as well as macrophage colony–stimulating factor (M-CSF), a key cytokine involved in macrophage differentiation that enhances the inflammatory response of primed macrophages (42). Interestingly, after 48 hours of mAb infusion, the only cytokines observed to be significantly altered (*n* = 8) were those that were also significantly altered on D0 (Figure 6B). By D7, several of these mutation-associated cytokines stayed altered (Figure 6C). These data suggest that, firstly, therapeutic mAbs do not substantially alter cytokine profiles in mildly ill COVID-19 patients, and secondly, cytokines identified to be linked to de novo S RBD mutation development are quite robust.

AUROC data were further validated with random forest classification, which identified a signature consisting of SAA, Tie-2, bFGF, and M-CSF that correctly identified patients with de novo S RBD mutations with high predictability (mean ROC of 96%). While C-reactive protein (CRP) on its own missed statistical significance with AUROC analysis, replacing CRP with SAA did not change the accuracy of the model, likely because of high degree of colinearity identified between CRP and SAA (Pearson’s *r* = 0.937, *P* < 0.001; Figure 6D). This signature was further independently tested on 19 patients, 8 of whom received sotrovimab and 11 of whom received tixagevimab/cilgavimab. Patient characteristics are described in Supplemental Figure 6A. One patient each receiving sotrovimab or tixagevimab/cilgavimab developed S RBD mutations within 7 days of receiving mAb therapy. All 19 samples were correctly classified utilizing the CIB-based signature, both by random forest classification (AUROC = 1) or a binomial logistic regression model (χ^2^ = 12.787, *n* = 19, df = 4, *P* < 0.012; Supplemental Figure 6, B and C). Remarkably, bFGF levels alone led to a 100% correct classification, with mutation carriers having bFGF levels of 23.7 pg/mL or higher (*n* = 2, range 23.7–34.4 pg/mL) and non–mutation carriers with levels of 19 pg/mL or lower (*n* = 17, average 5.5 pg/mL, range 0.5–19 pg/mL). These data not only suggest that a diminished proinflammatory and homeostatic cytokine immune milieu could facilitate development of de novo S RBD mutations, but also describe a CIB profile present before mAb administration that predicts development of escape mutations against therapeutic mAbs for SARS-CoV-2 in high-risk patients with high accuracy.

## Discussion

Absence of virus from respiratory tract samples is suggested to occur once serum neutralizing antibody titers of 1:80 or 2,000 BAU/mL are achieved (33, 43). Considering that the average serum antibody titers in mAb-treated patients are more than 1 million BAU/mL, or 1,000-fold higher than “high seropositivity” as defined by the WHO International SARS-CoV-2 Antibody Standards, our data suggest that therapeutic mAbs are unable to readily cross the respiratory mucosal barrier and neutralize SARS-CoV-2. All therapeutic mAbs investigated in this study are IgG subtypes, and while special mechanisms such as receptor-mediated IgG transport exist, most of the mucosal humoral immunity is either mediated by IgA or extravasated plasma cells that then locally secrete immunoglobins, including IgG (44–46). These data suggest that while therapeutic neutralizing mAbs efficiently clear SARS-CoV-2 from systemic tissue and reduce the risk of severe disease, the virus continues to thrive in the epithelial cells and mucosal barrier of the respiratory tract. With immunocompromised individuals exhibiting 4-fold higher viral loads compared with immunocompetent COVID-19 patients, these data not only support the evidence that immunocompromised patients have prolonged SARS-CoV-2 shedding (5, 7), but also suggest that innate cellular immunity is decisively involved in SARS-CoV-2 clearance from the respiratory tract. Our study design where patients received exogenous immunoglobins without affecting host plasma cells also offers insights into the relatively high importance of local secretion of immunoglobins by mucosal plasma cells, as opposed to transepithelial transport of immunoglobins, in conferring mucosal immunity. These data can also be extrapolated to humoral mucosal immunity against other respiratory viral and bacterial pathogens.

Not only do we show that respiratory viral carriage is more abundant in immunocompromised patients, we also show that occurrence of de novo mutations is significantly higher in these patients, as previously shown for severely or chronically ill immunocompromised COVID-19 patients (8–12). Most mutations in SARS-CoV-2 are either deleterious or relatively neutral and only a small proportion impact viral characteristics like transmissibility, virulence, and/or resistance to existing host immunity (1, 47). Concerns have also been raised that mutation rates could be overestimated due to reverse transcriptase or sequencing errors (11, 48). However, for the following reasons we believe that the identified mutations in S RBD are existent and nonneutral. First, potentially novel and unusually clustered mutations were reconfirmed by performing sequencing on independently extracted RNA, making it a high-fidelity observation. Second, S RBD mutations were identified 2–7 days after mAb treatment, in contrast to studies where mutations were observed before treatment, for example, case studies where mutations in S were fixed before casirivimab/imdevimab treatment (8, 49). Third, observed de novo mutations are highly specific to cognate mAb or ACE2 binding sites or its immediate proximity. For example, S RBD mutations developing in bamlanivimab- or bamlanivimab/etesevimab-treated patients had no overlap with mutated sites observed in sotrovimab-treated patients. Fourth, the de novo mutations are also highly evasive to therapeutic antibodies. For example, sotrovimab given empirically to BA.2-infected patients, against which sotrovimab shows little neutralization, did not lead to development of escape mutations, while it did for BA.1-infected patients against which sotrovimab is highly active. Fifth, sotrovimab-receiving BA.1-infected patients had more robust SARS-CoV-2–specific Th cell immunity, likely due to lack of SARS-CoV-2 neutralization. And, lastly, possible non-neutrality of some mutations described in this study are supported by prior reports on identical or similar mutations (25–31) (see Supplemental Table 4). Amino acid residues typically observed in Omicron subvariants reverting back to those of the original Wuhan-Hu-1 sequence (D339G, L371S, P373S, F375S, N417K, and K440N) are equally interesting, some of which have also been observed previously (17), supporting our seroneutralization data showing that sotrovimab is not active against Wuhan and some of the other de-escalated variants.

While we show that the de novo S RBD mutations are unequivocally mAb specific, we also show that mutations accumulate in acutely infected patients and occur rather rapidly, within 7 days of treatment. Prior studies have proposed that selection pressure created during chronic or severe infections drives the emergence of SARS-CoV-2 mutations (8–12). Our data suggest that neither the chronic nature of the disease nor its severity are necessary for occurrence of mutations if immune pressure is profound and rapid, as is that induced by synthetic neutralizing mAb therapy. Both RNA and DNA viruses are capable of generating de novo diversity in a short period of time while adapting to new hosts and environments (50). One thing common to both our and previous studies is that the mutation rate is significantly higher in immunocompromised patients (8–12); however, we also show that higher viral loads, regardless of the cause, are directly linked to S RBD mutation development.

We identify 2 specific components of host immunity that are associated with these mutations. Firstly, we demonstrate that downregulated proinflammatory cytokines are linked with higher rates of mutation, likely due to decreased viral clearance and more replication cycles giving the virus a higher chance to adapt evolutionarily. Cytokine immunity is an important component of innate and adaptive host immunity, and while examples exist where proinflammatory cytokines could be suppressed by viruses (51), the cytokine changes associated with de novo mutations are likely driven by host-genetic susceptibilities to SARS-CoV-2 (52). Secondly, in a non–mutually exclusive independent mechanism, we also show that patients developing de novo mutations had stronger Th cell immunity following mAb treatment, suggesting strong immune pressure on the virus to adapt (6). Additionally, we describe an upregulation of key host growth factors, such as angiogenic growth factors and their receptors, that could be a consequence of SARS-CoV-2–induced lung damage. However, because patients only had mild disease, we propose that a reparative milieu, likely also genetically driven, while facilitating a rapid recovery of patients could also allow boosted cell infection cycles that enable the virus to adapt. Our pharmacokinetic studies further showed that levels of all mAbs were maintained at more than 1 million BAU/mL over 4 weeks, suggesting a sustained longstanding environment wherein mutant SARS-CoV-2 could be sheltered and mutate further, posing threats for viral rebound infections and dissemination of novel mutants. It is hypothesized that almost all SARS-CoV-2 variants originated in immunocompromised chronic carriers (53). Our data therefore emphasize the need for optimized mitigation strategies in immunocompromised patients receiving mAb treatment to reduce the risk of SARS-CoV-2 spreading to other high-risk patients in both a hospital and community setting.

Lastly, we suggest that assessment of CRP or SAA (general marker of inflammation), bFGF (angiogenic ligand), Tie-2 (angiogenic growth factor receptor), and M-CSF (proinflammatory and immunoregulatory mediator) in high-risk patients with SARS-CoV-2 infection under evaluation for mAb therapy could identify patients, with high predictability, who are also at risk of developing escape mutations against therapeutic mAbs. This or similar biomarker-based stratification could also benefit decision making. For example, identification of immunocompromised patients who are also at high risk of developing de novo mutations could benefit from alternative strategies such as antiviral treatments or convalescent plasma containing high titers of polyclonal antibodies (54–56).

As limitations, samples analyzed in this study were collected during an extended time period, resulting in underlying differences in the patient population, such as rate of vaccination and circulating SARS-CoV-2 variants. At the same time, the heterogeneity of infecting VOCs and inclusion of vaccinated individuals among high-risk groups could be considered a strength of the study, as this enables representation of real-world data and rapid changes in epidemiological scenarios typical of the SARS-CoV-2 pandemic. Being a prospective monocentric cohort within a European project, this study had the advantage of homogeneous sampling and enrollment protocols, but lacks external validity. Finally, a very limited number of nasopharyngeal swab samples were collected on D28, thereby not allowing us to study the impact of mutation on prolonged carriage.

Despite these limitations, we show in a comprehensive analysis of patients with diverse mAb treatments development of adaptive mutations that highly correlate with neutralizing capacities of therapeutic mAbs, and provide direct evidence that anti–SARS-CoV-2 host-driven responses are necessary and essential for development of mutant SARS-CoV-2. While these data, on one hand, suggest a critical balance between successful viral killing and development of VOC-like mutations in niched environments such as respiratory mucosa, on the other hand our data also prompt close and extensive monitoring, and isolation of patients and contacts to limit the spread of potential VOC-like mutants, especially in high-risk populations.

## Methods

### Study design.

Samples were collected as part of the prospective, observational, monocentric ORCHESTRA cohort study conducted from March 9, 2021 to November 30, 2022 in the early COVID-19 treatment Outpatient Clinic, Infectious Diseases Section of the University Hospital of Verona, Italy. All outpatients aged 18 years or older, presenting with mild-to-moderate COVID-19 (confirmed by RT-qPCR or a positive antigenic third-generation test) at high risk for clinical worsening in accordance with Italian Medicine Agency indications (for definition see refs. 19, 20) were offered mAb therapy and enrolled in this study. All enrolled patients received treatment with bamlanivimab, bamlanivimab/etesevimab, casirivimab/imdevimab, or sotrovimab. In addition, a limited number of patients receiving tixagevimab/cilgavimab (*n* = 11) were also enrolled for assessment of the CIB profile predictive of development of SARS-CoV-2 mutations. Inclusion/exclusion criteria for patient enrollment have been published (19, 20).

Samples were collected from enrolled patients to study the effect of mAb therapy on SARS-CoV-2 viral load, mutations induced by different mAbs, mAb kinetics, neutralization capacity of mAbs, cellular immunity, and CIB responses, which were analyzed within ORCHESTRA WP6. For each enrolled patient, 4 time points were analyzed: (i) D0, just prior to mAb infusion; (ii) D2, 2 ± 1 days after mAb infusion on D0; (iii) D7, 7 ± 2 days after mAb infusion on D0; and (iv) D28, 28 ± 4 days after mAb infusion. Nasopharyngeal swab, serum, and peripheral blood mononuclear cell (PBMC) samples were collected along with clinical data. An overview of sample numbers included for each analysis is available in Supplemental Figure 7.

### SARS-CoV-2 viral load and variant sequencing.

RNA was extracted using the MagMAX Viral/Pathogen II Nucleic acid kit on a KingFisher Flex Purification System (Thermo Fisher Scientific). RT-qPCR was performed using the TaqPath COVID-19 CE-IVD RT-PCR Kit (Thermo Fisher Scientific) on a QuantStudio 5 Real Time PCR instrument (384-well block, 5 colors; Thermo Fisher Scientific). Extracted RNA was subjected to automated cDNA conversion and multiplexed library preparation using the Illumina COVIDSeq Test kit on a Zephyr G3 NGS (PerkinElmer) and sequenced using the High Output Kit v2 on a NextSeq 500/550 instrument (Illumina Inc.). Identified single nucleotide polymorphisms (SNPs) were verified by Sanger sequencing. For detailed methods, refer to the supplemental material.

### Serology.

Blood was collected in 10 mL serum tubes (Vacutainer K2E, BD Biosciences) and serum samples were prepared within 3 hours of blood collection. Anti-N, anti-S, and anti-RBD SARS-CoV-2 IgG titers were measured in serum samples using a multiplexed panel (Meso Scale Discovery) and data provided in WHO-recommended BAU units. For detailed methods, refer to the supplemental material.

### ACE2 neutralization measurements in serum.

ACE2 neutralization was measured in serum samples against Wuhan-Hu-1, Alpha/B.1.1.7, Omicron/BA.1, Omicron/BA.1+R346K, and Omicron/BA.2 using V-PLEX SARS-CoV-2 panels 6, 13, 23, and 25 (ACE2) on the QuickPlex SQ 120 system (Meso Scale Discovery) according to the manufacturer’s instructions. Further details regarding the S variants, against which the neutralizing antibody titers were measured, are displayed in Supplemental Table 6. For detailed methods, refer to the supplemental material.

### Measurements of CIBs in serum.

CIBs were measured in serum samples using U-plex and V-plex panels (K15198D and K15190D) on the QuickPlex SQ 120 system (Meso Scale Discovery), according to the manufacturer’s instructions. The following 40 CIBs were measured for the D0, D2, and D7 time points: bFGF, CRP, cutaneous T cell–attracting chemokine (CTACK), eotaxin, erythropoietin (EPO), Flt-1, fractalkine, M-CSF, IFN-β, IFN-γ, IL-1β, IL-1 receptor antagonist (IL-1Ra), IL-2, IL-2 receptor α (IL-2Rα), IL-4, IL-5, IL-6, IL-8, IL-10, IL-13, IL-15, IL-17A, IL-17F, IL-18, IL-22, IL-33, IFN-γ–induced protein 10 (IP-10), MCP-1, MCP-2, MCP-3, macrophage inflammatory protein 1α (MIP-1α), PlGF, SAA, soluble intercellular adhesion molecule 1 (sICAM-1), soluble vascular cell adhesion molecule 1 (sVCAM-1), Tie-2, tumor necrosis factor α (TNF-α), VEGF-A, VEGF-C, and VEGF-D. A small panel of 4 select CIBs, consisting of CRP, bFGF, Tie2, and M-CSF, was additionally utilized for validating CIB profile predictives of SARS-CoV-2 mutations. For detailed methods, refer to the supplemental material.

### SARS-CoV-2–specific cellular responses.

PBMCs were isolated using cellular preparation tubes (BD Biosciences) according to the manufacturer’s instructions and stored in fetal bovine serum (FBS) with 10% DMSO at –80°C until further use. Stimulation and staining were performed using the SARS-CoV-2 T Cell Analysis Kit (PBMC), human (Miltenyi Biotec). PBMCs were stimulated with a pool of lyophilized peptides, consisting of 15-mer sequences covering the complete protein-encoding sequence of the SARS-CoV-2 S glycoprotein (GenBank MN908947.3, Protein QHD43416.1) and the complete sequence of the N phosphoprotein (GenBank MN908947.3, Protein QHD43423.2) from Miltenyi Biotec. For detailed methods, refer to the supplemental material.

### Flow cytometry.

After stimulation, staining of surface and intracellular antigens was carried out with the following fluorochrome-conjugated recombinant human IgG1 isotype antibodies (Miltenyi Biotec): CD3-APC REAfinity (clone REA613), CD4-VioBright-B515 REAfinity (clone REA623), CD8-VioGreen REAfinity (clone REA734), CD14-CD20-VioBlue REAfinity (clone REA599, clone REA780), IFN-*γ*-PE REAfinity (clone REA600), TNF-α-PE-Vio 770 REAfinity (clone REA656), and CD154-APCVio 770 REAfinity (clone REA238). Samples were captured on a NovoCyte Quanteon 4025 flow cytometer (Agilent) and analyzed using FlowJo v10.8.1 (BD Biosciences) (Supplemental Figure 8). For detailed methods, refer to the supplemental material.

### Data availability.

Data supporting the findings of this study are available within supplemental material files. SARS-CoV-2 genome sequences obtained in the project were submitted to GISAID (https://gisaid.org/). Trimmed read data generated and used for identification of emerging de novo S RBD mutants in this study have been submitted to the European Nucleotide Archive (ENA) under project accession no. PRJEB55794. All other data generated in this study are available from the corresponding author upon request.

### Statistics.

All data were statistically analyzed and visualized in Rstudio v.1.3.1073 (https://github.com/rstudio/rstudio) using R v.4.0.4 (https://www.r-project.org/). One-way analysis of variance (ANOVA) was utilized for longitudinal and cross-sectional comparisons of IgG titers, titers of neutralizing antibodies, and CIB concentrations across treatment groups followed by pairwise 2-tailed *t* tests. Ct values were compared using nonparametric Kruskal-Wallis test followed by pairwise testing using the Mann-Whitney test. Post hoc *P*-value correction was conducted using Bonferroni’s multiple-comparison correction method for all analyses. Throughout the statistical analyses, values below the detection range were recorded as 1/10 the lower limit of quantitation (LLQ) and values above the detection range were recorded as upper limit of quantitation (ULQ). A (corrected) *P* value of less than 0.05 was considered statistically significant. For the identification of the main predictors of qualitative responses (mutation/no mutation in the S RBD region [residues 319–541]), ROC curves were constructed utilizing MetaboAnalyst (https://dev.metaboanalyst.ca/MetaboAnalyst/). Machine learning–based random forest classifiers (RFCs) were further built by the Python package sklearn v3.10 (https://www.python.org/) to independently predict development of de novo S RBD mutations in patients receiving mAb regimens. Each model was built with a training set of values consisting of 70% of the data and a test set of 30% (57). To account for imbalanced groups, the synthetic minority oversampling technique (SMOTE, Python package imblearn 0.8.0) was utilized in combination with the RCF method. The models were bootstrapped 100 times and features for each model were selected based on (a) feature importance, (b) statistics from mutation versus nonmutation, (c) individual ROC curve analysis, and (d) a Pearson correlation matrix for independence of variables. Confusion matrices and ROC curves were drawn to calculate AUROC values to verify reliability and to evaluate the performance of the constructed models. The CIB model built to predict emergence of evasive SARS-CoV-2 S RBD mutations in patients treated with mAbs in the main study population was validated both by RFC and binomial logistic regression in a patient cohort on independently generated data sets. Linear mixed models were utilized to investigate evolution of antibody titers and Th cell immunity over time between the different mAb groups.

### Study approval.

Participants were recruited from the Infectious Diseases Section of the University Hospital of Verona from March 9, 2021 to November 30, 2022. All volunteers provided informed, written consent before study participation. This study was approved by the University Hospital Verona Ethics Board (protocol number: 19293) and conducted in accordance with the Declaration of Helsinki.

## Author contribution

SKS, ET, SMK, and PDN conceptualized the study. SKS provided overall study supervision. AS, PDN, MM, DP, ED, ER, and ET collected clinical data and samples. MS, MB, and SMK performed RT-qPCR and viral variant sequencing. MB and MS conducted bioinformatics analyses. AK, AG, and FHRDW performed serological analyses. AK, AG, FHRDW, and AH performed CIB analyses. DP, PD, and ER isolated PBMCs, which were analyzed by AG, VVA, AK, and FHRDW. MB, AG, AK, and SKS performed statistical analyses. SKS, SMK, ET, AG, AK, MB, and MS interpreted data. SKS, MB, AG, AK, MS, SMK, and ET wrote the manuscript. All authors read, gave input, and approved the final manuscript. The co–first authorship and order was determined by their contributions to the findings described in this manuscript.

## Supplementary Material

Supplemental data

ICMJE disclosure forms

## Figures and Tables

**Table 1:**
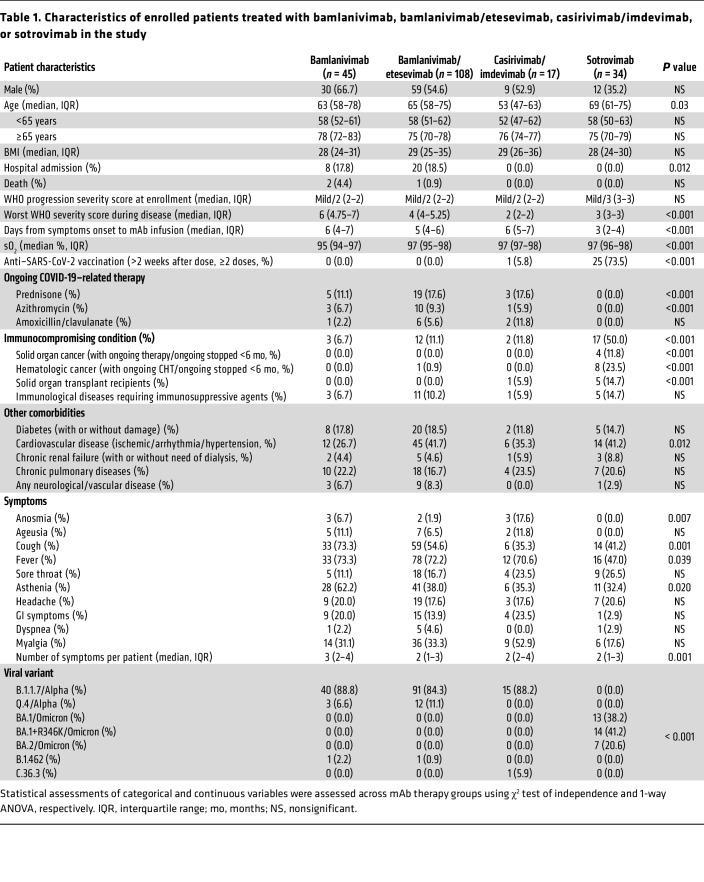
Characteristics of enrolled patients treated with bamlanivimab, bamlanivimab/etesevimab, casirivimab/imdevimab, or sotrovimab in the study

## Version 1 02/09/2023

In-Press Preview

## Version 2 03/15/2023

Electronic publication
