# Supplementary material for: Host immunological responses facilitate development of SARS-CoV-2 mutations in patients receiving monoclonal antibody treatments
Source: J Clin Invest. 2023 Mar 15;133(6):e166032. doi: 10.1172/JCI166032 (PMC10014108; doi:10.1172/JCI166032)
Supplement: Supplemental data [file jci-133-166032-s008.pdf]

Supplemental Information

Host immunological responses facilitate development of SARS-CoV-2 mutations in patients receiving monoclonal antibody treatments

Akshita Gupta[1,2*], Angelina Konnova[1,2*], Mathias Smet[1,2*], Matilda Berkell[1,2*], Alessia Savoldi[3], Matteo Morra[3], Vincent Van averbeke[1], Fien HR De Winter[1], Denise Peserico[4], Elisa Danese[4], An Hotterbeekx[1], Elda Righi[3], mAb ORCHESTRA working group, Pasquale De Nardo[3], Evelina Tacconelli[3], Surbhi Malhotra Kumar[2#], Samir Kumar Singh[1,2#†]

[1]Molecular Pathology Group, Cell Biology & Histology, Faculty of Medicine and Health Sciences, University of Antwerp, Antwerp, Belgium
[2]Laboratory of Medical Microbiology, Vaccine & Infectious Disease Institute, University of Antwerp, Antwerp, Belgium
[3]Division of Infectious Diseases, Department of Diagnostics and Public Health, University of Verona, Verona, Italy
[4]Section of Clinical Biochemistry, University of Verona, Verona, Italy

* Shared first authors
# Shared senior authors: samir.kumarsingh@uantwerpen.be; surbhi.malhotra@uantwerpen.be
† Correspondence: samir.kumarsingh@uantwerpen.be

Supplemental Methods

RNA extraction, cDNA conversion, library preparation, and SARS-CoV-2 whole genome sequencing

RNA was extracted using the MagMAX Viral/Pathogen II Nucleic acid kit on a KingFisher Flex Purification System (ThermoFisher). Each batch of samples taken forward for extraction was processed together with a Twist synthetic SARS-CoV-2 RNA positive Ctrl. 18 (Cat. No: 104338, Twist Bioscience). Extracted RNA was subjected to automated cDNA conversion and multiplexed library preparation using the Illumina COVIDSeq Test kit (Illumina Inc.) on a Zephyr G3 NGS system (PerkinElmer, MA, USA). DNA concentrations were quantified using the Qubit dsDNA HS Assay Kit (Invitrogen, Cat. No. Q33231) using a Qubit Fluorometer 3.0 (ThermoFisher). Pooled libraries were sequenced utilizing the High Output Kit v2 with a 1.4 nM PhiX Library positive control v3 using a 1% spike-in on a NextSeq 500/550 instrument (Illumina Inc.). All steps were performed according to manufacturer's instructions.

SARS-CoV-2 RT-qPCR

Real-Time RT-qPCR was performed using the TaqPath™ COVID-19 CE-IVD RT-PCR Kit (ThermoFisher) on a QuantStudio™ 5 Real Time PCR instrument (384-well block, 5 colors, ThermoFisher), which detects three genes in the SARS-CoV-2-viral genome: the S protein, N protein, and ORF1ab. MS2 (phage control) was added to each sample prior to RNA extraction to serve as internal control. RT-qPCR analysis was performed using FastFinder (UgenTec). Samples were considered positive if both the MS2 phage control (Ct < 32) and at least two gene targets were detected (Ct < 37).

SARS-CoV-2 variant detection

Raw sequencing data quality for each sample was assessed using FastQC (https://www.bioinformatics.babraham.ac.uk/projects/fastqc/) followed by quality trimming using a Phred score cut-off of 25 with TrimGalore v. 0.6.7 (https://github.com/FelixKrueger/TrimGalore). Read mapping was performed against the SARS-CoV-2 genome (GenBank: NC_045512.2) using the CLC Genomics Workbench v.9.5.3 (Qiagen) with a length and a similarity fraction of 0.5 and 0.8, respectively. Consensus sequences were extracted, and clade and lineage assignment performed using Nextstrain (https://clades.nextstrain.org/) and Pangolin (https://pangolin.cog-uk.io/), respectively. SARS-CoV-2 genome sequencing was considered successful if: i) was successfully classified by both Pangolin and NextClade, and ii) the resulting genome sequence harbored < 15% ambiguous base calls (Ns) in the consensus sequence.

For detection of single nucleotide variations (SNPs) acquired during monoclonal antibody treatment in patients who provided samples at D0, as well as D2 and/or D7, trimmed reads were mapped against the SARS-CoV-2 genome (GenBank: NC_045512.2) using the CLC Genomics Workbench v.9.5.3 (Qiagen) with a length and a similarity fraction of 0.7 and 0.99, respectively. SNPs resulting in amino acid substitutions were of particular interest and analyzed further in this study.

SNP validation using Sanger sequencing

Patients harboring non-synonymous mutations in the Spike RBD region (residues 319 – 541) were subjected to Sanger sequencing. For this purpose, RNA extraction and cDNA conversion was repeated and used for the validation.

Primers binding in the region of interest were selected from the Artic primer pool v3 (nCoV-2019 sequencing protocol v3 (LoCost) by performing in silico PCR using the CLC Genomics Workbench v.9.5.3 (Qiagen) or designed using NCBI PrimerBlast with standard parameters (National Center for Biotechnology Information) utilizing the Wuhan (GenBank: NC_045512.2) and Omicron/BA1.1

(GenBank: OM664849) genomes as templates with the following criteria: i) optimal primer length = 25 bp, ii) >5 bp difference in length between forward and reverse primers, and iii) ΔTm <5°C. Designed primer pairs were validated in silico using FastPCR (https://primerdigital.com/) using standard parameters.

PCR amplification was performed using 50 ng cDNA, Q5 Hot start 2x MM (New England Biolabs), forward and reverse primers at a final concentration of 0.5 µM each, and Nuclease-free water (Ambion, ThermoFisher) in a total volume of 45 µL with the following temperature profile: 98°C for 15s followed by 35 cycles of denaturation at 96°C for 30 s and annealing at 63°C for 5 min. Successful amplification was confirmed with 1.5% agarose gel electrophoresis (150 V, 200 mA, 1h) using a 100 bp DNA ladder (ThermoFisher).

Obtained PCR products were then subjected to automatic template clean-up and sample preparation using Illustra™ ExoProStar™ (Merck) and ABI PRISM® BigDye™ Terminator cycle sequencing kits (ThermoFisher) with Biomek® FX and NX liquid handlers (Tecan), followed by sequencing on an Applied Biosystems 3730XL DNA Analyzer (ThermoFisher). Sequence analysis was performed using the CLC Genomics Workbench v.9.5.3 (Qiagen).

Serology

Blood was collected in 10 mL serum tubes (BD vacutainer K2E, BD Biosciences) and serum samples were prepared within 3h of blood collection. Serum was allowed to clot thoroughly for 60 min before separation by centrifuging for 10 min at 1300 g. Aliquots were flash frozen in liquid nitrogen, shipped to the University of Antwerp for further processing and stored at -80°C until analysis.

IgG titers were measured in serum samples using the V-PLEX SARS-CoV-2 Panel 6 Kit (IgG; #K15433U-4) on a QuickPlex SQ 120 instrument (Meso Scale Discovery (MSD)) according to the manufacturer's instructions. IgG titers to the following antigens were measured: SARS-CoV-2 Nucleocapsid, SARS-CoV-2 S1 RBD, SARS-CoV-2 Spike, SARS-CoV-2 Spike (D614G), SARS-CoV-2 Spike (B.1.1.7/Alpha), SARS-CoV-2 Spike (B.1.351/Beta), SARS-CoV-2 Spike (P.1/Gamma). Baseline samples were measured at 1:1,000 or 1:10,000, while all other samples were measured at a final dilution of 1:10,000,000 or 1:100,000,000 in Diluent 100 (MSD). Quantitative IgG results were measured in Antibody Units (AU)/mL converted to Binding Antibody Units (BAU)/mL using a conversion factor provided by the manufacturer and reported as such. Patients were considered negative if their levels were under 4.76 BAU/mL for anti-spike, under 5.58 BAU/mL for anti-RBD, and under 8.20 BAU/mL for anti-nucleocapsid, these limits were determined by calculating the average plus one standard deviation of IgG measurements in 56 serum samples collected before 2019.

ACE2 neutralization measurements in serum

ACE2 neutralization measured in diluted serum samples (1:3,000) using V-PLEX SARS-CoV-2 Panel 6, 13, 23 and 25 (ACE2) and measured on the QuickPlex SQ 120 instrument (MSD) according to the manufacturer's instructions. Details regarding the Spike variants, against which the neutralizing antibody titers were measured, are displayed in **Supplemental Table 6**. Quantitative ACE2 neutralization results were measured in Units (U)/mL for all variants except Omicron sub-variants, which corresponds to neutralizing activity of 1 µg/mL monoclonal antibody to SARS CoV-2 Spike protein (upper limit of quantitation: 63,000 U/mL; lower limit of quantitation: 15 U/mL). Omicron sub-variants were measured as percent inhibition (% inhibition) calculated as 100 x [1 - (Sample signal/Average signal of the blanks)].

SARS-CoV-2 specific cellular responses

Peripheral blood mononuclear cells (PBMCs) were isolated using cellular preparation tubes (BD Biosciences, Germany) according to the manufacturer's instructions and frozen in fetal bovine serum (FBS) with 10% DMSO until further use. Stimulation and staining were performed using the SARS-

CoV-2 T Cell Analysis Kit (PBMC) human (Miltenyi Biotech). Briefly, PBMCs were thawed and rested overnight in RPMI 1640 medium (Gibco, ThermoFisher, the Netherlands) supplemented with 5% heat-inactivated AB serum (Sigma-Aldrich, Merck), 100 U/ml penicillin (Biochrom), and 0.1 mg/ml streptomycin (Biochrom). 1e6 PBMCs were stimulated with a pool of lyophilized peptides, consisting mainly of 15-mer sequences with 11 amino acids overlap, covering the complete protein coding sequence (residues 5–1273) of the SARS-CoV-2 surface or Spike glycoprotein of SARS-CoV-2 (GenBank MN908947.3, Protein QHD43416.1) and the complete sequence of the nucleocapsid phosphoprotein (GenBank MN908947.3, Protein QHD43423.2) from Miltenyi Biotech. Both peptide pools were used at 1 µg/mL per peptide. Stimulation controls were performed with equal concentrations of sterile water/10% DMSO (unstimulated) as negative control and Cytostim (Miltenyi Biotech) as positive control. Incubation was performed at 37 °C, 5% CO2 for 6h with 2 µg/mL brefeldin A (Sigma-Aldrich, Merck) added after 2 h.

Flow cytometry

After stimulation, staining of surface and intracellular antigens was carried out with the following fluorochrome-conjugated recombinant human IgG1 isotype antibodies (Miltenyi Biotech) at 0.25x recommended volume: CD3-APC REAfinity (clone REA613), CD4-Vio Bright-B515 REAfinity (clone REA623), CD8-VioGreen REAfinity (clone REA734), CD14-CD20-VioBlue REAfinity (clone REA599, clone REA780), IFN-γ-PE REAfinity (clone REA600), TNF-α-PE-Vio 770 REAfinity (clone REA656), CD154-APCVio 770 REAfinity (clone REA238). Cells were washed with cell staining buffer (PBS 1% bovine serum albumin, 2mM EDTA) unless stated otherwise. Briefly, dead cells were stained for 10 min with Viobility 405/452 Fixable Dye (1:200) with subsequent fixation and permeabilization for 20min (Inside stain kit, Miltenyi Biotech). Cells were washed with permeabilization buffer and surface marker, and intracellular staining was carried out for 15 min. Cells were washed in permeabilization buffer and resuspended in cell staining buffer. Samples were captured on a NovoCyte Quanteon 4025 flow cytometer (Agilent) and analyzed using FlowJo v10.8.1 (BD) (**Supplemental Figure 8**).

Supplemental Tables

Supplemental Table 1. Eligibility criteria. Italian Medicines Agency Emergency Use Authorization eligibility criteria for bamlanivimab, bamlanivimab/etesevimab, casirivimab/imdevimab, and sotrovimab therapy in adult patients as described by Savoldi et al.(2, 3).

**Patients enrolled during March 2021 – 15 June 2021:**
**All the following criteria should be met:**<br>1. Confirmed diagnosis of SARS-CoV-2 infection either by polymerase chain reaction or 3rd generation antigenic test on nasopharyngeal swab<br>2. Onset of at least one of the COVID-19 related symptoms among fever, cough, dyspnea, headache, myalgia, gastrointestinal symptoms, asthenia ≤ 10 days<br>3. Age ≥ 18 years<br>4. Body weight ≥40 kg<br>5. No need for oxygen therapy<br>6. No need for hospitalization<br>7. Presence of at least one of the following medical conditions:<br>• BMI ≥ 35 Kg/m^2<br>• Subject chronically undergoing peritoneal dialysis or hemodialysis<br>• Uncontrolled diabetes mellitus (HbA1c ≥ 9% or 75 mmol/L) or with chronic complications<br>• Primary immunodeficiency<br>• Secondary immunodeficiency (e.g., hematologic cancer patient in ongoing myeloid/immunosuppressive therapy or suspension for <6 months)<br>• Cardio-cerebrovascular disease (including arterial hypertension with documented organ damage) in subjects aged ≥ 55 years<br>• Chronic Obstructive Pulmonary Disease and/or other chronic respiratory disease in subjects ≥ 55 years
**Patients enrolled during 16 June – ongoing:**
**All the following criteria should be met:**<br>1. Confirmed diagnosis of SARS-CoV-2 infection either by polymerase chain reaction or 3rd generation antigenic test on nasopharyngeal swab<br>2. Onset of at least one of the COVID-19 related symptoms among fever, cough, dyspnea, headache, myalgia, gastrointestinal symptoms, asthenia ≤ 7 days<br>3. Age ≥ 18 years<br>4. Body weight ≥40 kg<br>5. No need for oxygen therapy<br>6. No need for hospitalization<br>7. Presence of at least one of the following medical conditions:<br>• Age > 65 years<br>• BMI ≥ 30 Kg/m2<br>• Chronic kidney disease (including dialysis)<br>• Uncontrolled diabetes mellitus (HbA1c ≥ 9% or 75 mmol/L) or with chronic complications<br>• Primary immunodeficiency<br>• Secondary immunodeficiency (e.g., hematologic cancer patient in ongoing myeloid/immunosuppressive therapy or suspension for <6 months)<br>• Cardio-cerebrovascular disease (including arterial hypertension with documented organ damage)<br>• Chronic Obstructive Pulmonary Disease and/or other chronic respiratory disease<br>• Chronic liver disease<br>• Hemoglobinopathies<br>• Neurodevelopmental diseases and neurodegenerative diseases

Supplemental Table 2. Results of real-time reverse transcriptase quantitative (RT-)qPCR detection of the *ORF1ab*, *N*, and *S* protein genes in nasopharyngeal swab samples collected from patients treated with bamlanivimab, bamlanivimab/etesevimab, casirivimab/imdevimab, or sotrovimab. Statistical comparisons between treatment groups at each timepoint were performed using Kruskal-Wallis. Ct: cyclic threshold. CI: confidence interval. Only Ct values for variants of concern were considered. Positive samples collected at day 28 post mAb infusion were limited (2/14) and were therefore excluded from this analysis. D0: sample collected prior to mAb infusion. D2: 2 ± 1 days after mAb infusion. D7: 7 ± 2 days after mAb infusion.

	D0			D2			D7		
**Treatment**	**n**	**Ct average (95% CI)**	**p-value**	**n**	**Ct average (95% CI)**	**p-value**	**n**	**Ct average (95% CI)**	**p-value**
ORF1ab									
Bamlanivimab	44	19.25 (17.8 - 20.7)	0.008	33	24.33 (22.8 - 25.8)	2.20E-07	16	26.14 (23.9 - 28.4)	0.335
Bamlanivimab/etesevimab	107	19.31 (18.4 - 20.2)		80	23.21 (22.4 - 24.0)		43	26.62 (25.7 - 27.5)	
Casirivimab/imdevimab	17	19.29 (17.2 - 21.4)		15	24.11 (21.9 - 26.3)		5	27.84 (26.1 - 29.6)	
Sotrovimab	34	16.35 (15.2 - 17.5)		33	19.02 (17.7 - 20.4)		34	24.49 (22.6 - 26.4)	
S protein									
Bamlanivimab	0	-	-	0	-	-	0	-	-
Bamlanivimab/etesevimab	0	-		0	-		0	-	
Casirivimab/imdevimab	0	-		0	-		0	-	
Sotrovimab	7	14.94 (12.7 - 17.2)		7	17.09 (13.6 - 20.6)		6	23.40 (19.8 - 27.0)	
N protein									
Bamlanivimab	44	18.18 (16.8 - 19.5)	0.108	33	22.58 (21.2 - 24.0)	2.70E-05	16	24.28 (22.1 - 26.4)	0.686
Bamlanivimab/etesevimab	107	18.18 (17.3 - 19.1)		80	21.38 (20.5 - 22.2)		43	25.08 (24.3 - 25.9)	
Casirivimab/imdevimab	17	18.66 (16.6 - 20.8)		15	22.82 (20.7 - 25.0)		5	26.38 (23.8 - 29.0)	
Sotrovimab	34	16.06 (14.9 - 17.2)		33	18.51 (17.3 - 19.7)		34	23.96 (22.1 - 25.8)	

Supplemental Table 3. Results of real-time reverse transcriptase quantitative (RT-)qPCR detection of the *ORF1ab*, *N*, and *S* protein genes in nasopharyngeal swab samples collected from patients with different variants – Alpha and Omicron sub-variants. Statistical comparisons between treatment groups at each timepoint were performed using Kruskal-Wallis. Ct: cyclic threshold. CI: confidence interval. Positive samples collected at day 28 post mAb infusion were limited (2/14) and were therefore excluded from this analysis. D0: sample collected prior to mAb infusion. D2: 2 ± 1 days after mAb infusion. D7: 7 ± 2 days after mAb infusion.

	D0			D2			D7		
	n	Ct average (95% CI)	p-value	n	Ct average (95% CI)	p-value	n	Ct average (95% CI)	p-value
**ORF1ab**									
Alpha	161	19.3 (18.6 – 20.0)	2.10E-04	123	23.5 (22.8 – 24.2)	1.20E-08	62	26.5 (25.7 – 27.3)	0.057
Omicron	34	15.1 (14.0 – 17.9)		33	18.6 (16.4 – 21.4)		34	23.6 (19.5 – 29.0)	
B.1.1.7	146	19.4 (18.6 – 20.2)	9.60E-04	110	23.5 (22.8 – 24.3)	4.80E-07	56	26.9 (26.1 – 27.6)	0.096
Q.4	15	18.5 (16.2 – 20.9)		13	23.4 (20.2 – 26.7)		6	22.8 (17.8 – 27.9)	
BA.1	13	18.2 (15.8 – 20.6)		13	20.1 (17.3 – 22.9)		13	25.0 (21.9 – 28.1)	
BA.1+R346K	14	15.3 (14.0 – 16.7)		13	18.9 (17.2 – 20.6)		14	23.9 (20.4 – 27.4)	
BA.2	7	14.8 (14.1 – 15.1)		7	16.1 (15.1 – 19.7)		7	23.4 (21.7 – 27.3)	
**N gene**									
Alpha	161	18.2 (17.5 – 18.9)	0.008	123	21.7 (21.1 – 22.4)	5.40E-06	62	24.9 (24.1 – 25.6)	0.375
Omicron	34	15.3 (13.5 – 18.3)		33	18.2 (16.0 – 20.7)		34	23.0 (19.7 – 28.4)	
B.1.1.7	146	18.3 (17.5 – 19.1)	0.023	110	21.8 (21.1 – 22.5)	1.20E-04	56	25.3 (24.6 – 25.9)	0.248
Q.4	15	17.4 (15.1 – 19.7)		13	21.7 (18.7 – 24.6)		6	21.2 (16.5 – 25.9)	
BA.1	13	17.7 (15.3 – 20.2)		13	19.3 (17.0 – 21.7)		13	24.4 (21.3 – 27.5)	
BA.1+R346K	14	15.3 (13.8 – 16.8)		13	18.6 (17.0 – 20.2)		14	23.5 (20.0 – 27.0)	
BA.2	7	14.4 (13.5 – 14.6)		7	15.4 (14.4 – 19.4)		7	23.0 (20.8 – 26.8)	

Supplemental Table 4. De novo SARS-CoV-2 variants emerging during the first seven days of monoclonal antibody treatment. Only non-synonymous mutations detected at D2 or D7 compared to D0 are reported. All reference positions refer to the Wuhan variant (GenBank: NC_045512.2). *: deletion. †: variant of concern mutation emerging irrespective of mAb therapy. Fs: frameshift. D0: sample collected prior to mAb infusion. D2: 2 ± 1 days after mAb infusion. D7: 7 ± 2 days after mAb infusion.

Reference position	ORF/gene	Amino acid substitution	Previously reported mutations in the same codon	References
701	ORF1ab	G146S	—	—
1478	ORF1ab	A405S	—	—
2841	ORF1ab	V859A	—	—
2864	ORF1ab	E867*	—	—
4592	ORF1ab	E1443*	—	—
6456	ORF1ab	C2064Y	—	—
6615	ORF1ab	L2117S	—	—
6843	ORF1ab	S2193F	—	—
7860	ORF1ab	T2532I	—	—
7987	ORF1ab	Q2574H	—	—
8505	ORF1ab	T2747I	—	—
11490	ORF1ab	S3742F	—	—
12067	ORF1ab	M3934I	—	—
13065	ORF1ab	L4267S	—	—
14503	ORF1ab	H4747Y	—	—
16795	ORF1ab	V5511L	—	—
18551	ORF1ab	S6096T	—	—
21458	ORF1ab	I7065T	—	—
22484	S	V308L	—	—
22578	S	D339G	G339D †	(4)
22580	S	E340K	E340K/A/D/G/Q	(5-7)
22581	S	E340V	E340K/A/D/G/Q	(5-7)
22582	S	E340D	E340K/A/D/G/Q	(5-7)
22599	S	K346R	R346K/T/S/M	(8)
22673	S	L371S	S371L, L371S †	(4, 9)
22679	S	P373S	S373P, P373S †	(4, 9)
22686	S	F375S	F375S †	(9)
22813	S	N417K	K417N/T †	(4, 5, 10, 11)
22882	S	K440N	N440K	(8)
23012	S	E484K	E484K/Q	(8, 12-15)
23013	S	E484A	—	—
23014	S	E484D	—	—
23039	S	Q493K	Q493K	(8, 10, 11, 15)
23040	S	Q493R	Q493R	(8, 10, 11, 15)
23042	S	S494P	S494P	(12, 14)
23401	S	Q613H	—	—
23709	S	I716T	—	—
24029	S	F823I	—	—
24939	S	C1126F	—	—
25024	S	Y1155fs	—	—
25407	ORF3a	M5I	—	—
25784	ORF3a	W131L	—	—
25811	ORF3a	L140P	—	—
27145	M	T208I	—	—
27462	ORF7a	C23W	—	—
27610	ORF7a	H73Y	—	—
27874	ORF7b	T40I	—	—
28987	N	Q239fs	—	—

Supplemental Table 5. Distribution of patients tested for serological analysis among different treatment groups. Number and percentage of patients receiving bamlanivimab, bamlanivimab/etesevimab, casirivimab/imdevimab, or sotrovimab therapy that were fully vaccinated (14 days after second vaccination dose) or unvaccinated.

Therapy	Bamlanivimab			Bamlanivimab/etesevimab			Casirivimab/imdevimab			Sotrovimab		
**Unvaccinated**	**n = 45**			**n = 108**			**n = 16**			**n = 10**		
	**Spike**	**RBD**	**Nucleocapsid**	**Spike**	**RBD**	**Nucleocapsid**	**Spike**	**RBD**	**Nucleocapsid**	**Spike**	**RBD**	**Nucleocapsid**
Negative	30 (66.6%)	34 (75.5%)	36 (80.0%)	88 (81.5%)	90 (83.3%)	98 (90.7%)	12 (75%)	11 (68.8%)	13 (81.3%)	1 (10%)	3 (30%)	7 (70%)
Inconclusive	9 (9.2%)	7 (15.5%)	3 (6.6%)	18 (16.7%)	15 (13.9%)	2 (1.9%)	2 (12.5%)	2 (12.5%)	0 (0%)	6 (60%)	3 (30%)	0 (0%)
Positive – Low	2 (4.4%)	3 (6.6%)	4 (8.8%)	2 (1.9%)	2 (1.9%)	8 (7.4%)	0 (0%)	2 (12.5%)	3 (18.6%)	2 (20%)	3 (30%)	3 (30%)
Positive – Medium	1 (4.4%)	0 (0%)	0 (0%)	0 (0%)	1 (0.9%)	0 (0%)	2 (12.5%)	1 (6.2%)	0 (0%)	0 (0%)	1 (10%)	0 (0%)
Positive – High	0 (0%)	0 (0%)	0 (0%)	0 (0%)	0 (0%)	0 (0%)	0 (0%)	0 (0%)	0 (0%)	1 (10%)	1 (10%)	0 (0%)
**Vaccinated**	**n = 0**			**n = 0**			**n = 1**			**n=25**		
	**Spike**	**RBD**	**Nucleocapsid**	**Spike**	**RBD**	**Nucleocapsid**	**Spike**	**RBD**	**Nucleocapsid**	**Spike**	**RBD**	**Nucleocapsid**
Negative	0 (0%)	0 (0%)	0 (0%)	0 (0%)	0 (0%)	0 (0%)	0 (0%)	0 (0%)	1 (100%)	3 (12%)	2 (8%)	22 (88%)
Inconclusive	0 (0%)	0 (0%)	0 (0%)	0 (0%)	0 (0%)	0 (0%)	0 (0%)	0 (0%)	0 (0%)	3 (12%)	4 (16%)	1 (4%)
Positive – Low	0 (0%)	0 (0%)	0 (0%)	0 (0%)	0 (0%)	0 (0%)	1 (100%)	1 (100%)	0 (0%)	1 (4%)	1 (4%)	2 (8%)
Positive – Medium	0 (0%)	0 (0%)	0 (0%)	0 (0%)	0 (0%)	0 (0%)	0 (0%)	0 (0%)	0 (0%)	0 (0%)	0 (0%)	0 (0%)
Positive – High	0 (0%)	0 (0%)	0 (0%)	0 (0%)	0 (0%)	0 (0%)	0 (0%)	0 (0%)	0 (0%)	18 (72%)	18 (72%)	0 (0%)

Supplemental Table 6. SARS-CoV-2 Spike antigens of variants of concern (VOCs) tested by ACE2 seroneutralization in relation to the wild-type (Wuhan) SARS-CoV-2 variant. Amino acid modification and commonly used variant of concern (VOC) designations are summarized as described for the utilized V-PLEX Serology Panel (Meso Scale Discovery (MSD)) for VOCs and variants of interest used in this study.

**Lineages**	**Amino Acid Modifications**	**Common Designation**
B.1.1.7	ΔH69-V70, ΔY144, N501Y, A570D, D614G, P681H, T716I, S982A, D1118H	Alpha
B.1.1.529; BA.1; BA.1.15	A67V, ΔH69-V70, T95I, G142D, Δ143-145, Δ211/L212I, ins214EPE, G339D, S371L, S373P, S375F, K417N, N440K, G446S, S477N, T478K, E484A, Q493R, G496S, Q498R, N501Y, Y505H, T547K, D614G, H655Y, N679K, P681H, N764K, D796Y, N856K, Q954H, N969K, L981F	Omicron sub-lineage
B.1.1.529; BA.1+R346K; BA.1.1; BA.1.1.15	A67V, Δ69-70, T95I, G142D/Δ143-145, Δ211/L212I, ins214EPE, G339D, R346K, S371L, S373P, S375F, K417N, N440K, G446S, S477N, T478K, E484A, Q493R, G496S, Q498R, N501Y, Y505H, T547K, D614G, H655Y, N679K, P681H, N764K, D796Y, N856K, Q954H, N969K, L981F	Omicron sub-lineage
B.1.1.529; BA.2; BA.2.1; BA.2.2; BA.2.3; BA.2.5; BA.2.6; BA.2.7; BA.2.8; BA.2.10; BA.2.12	T19I, (L24-A27)toS, G142D, V213G, G339D, S371F, S373P, S375F, T376A, D405N, R408S, K417N, N440K, S477N, T478K, E484A, Q493R, Q498R, N501Y, Y505H, D614G, H655Y, N679K, P681H, N764K, D796Y, Q954H, N969K	Omicron sub-lineage

Supplemental Figures

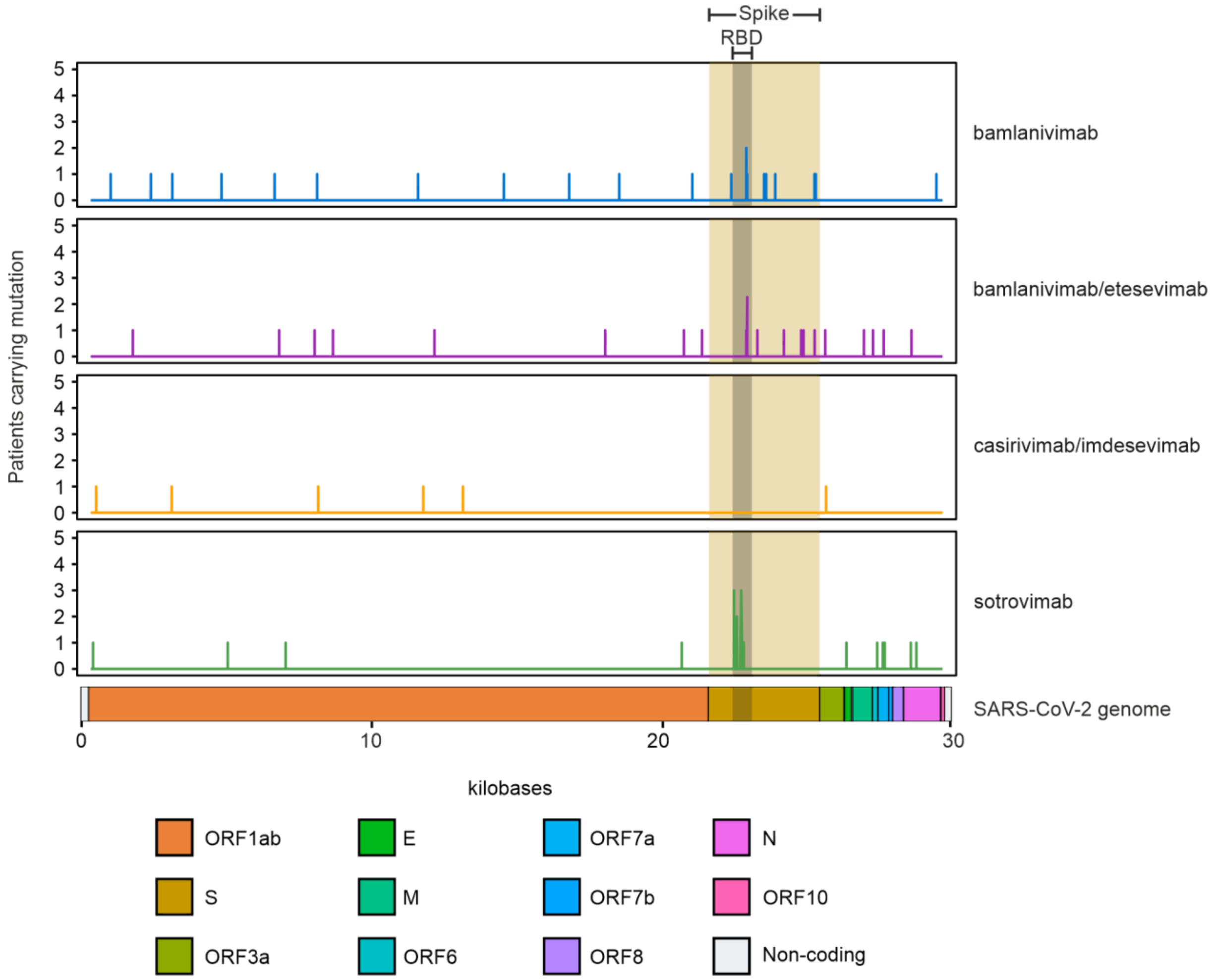

Supplemental Figure 1. Patients receiving mAb treatment develop non-synonymous de novo mutations in the SARS-CoV-2 genome two (D2) to seven days (D7) after mAb infusion. Number of events of unique de novo mutations identified at D2 or D7 compared to D0 (baseline) are plotted across the positions in the SARS-CoV-2 genome. The number of patients developing mutations at specific positions in the SARS-CoV-2 genome are displayed for patients receiving bamlanivimab, bamlanivimab/etesevimab, casirivimab/imdevimab, or sotrovimab in the study. Only non-synonymous mutations are indicated in the figure. The Spike gene is highlighted in yellow, whereas Spike RBD is indicated in gray. D0: sample collected prior to mAb infusion. D2: 2 ± 1 days after mAb infusion. D7: 7 ± 2 days after mAb infusion. For more details, see **Supplemental Table 4**.

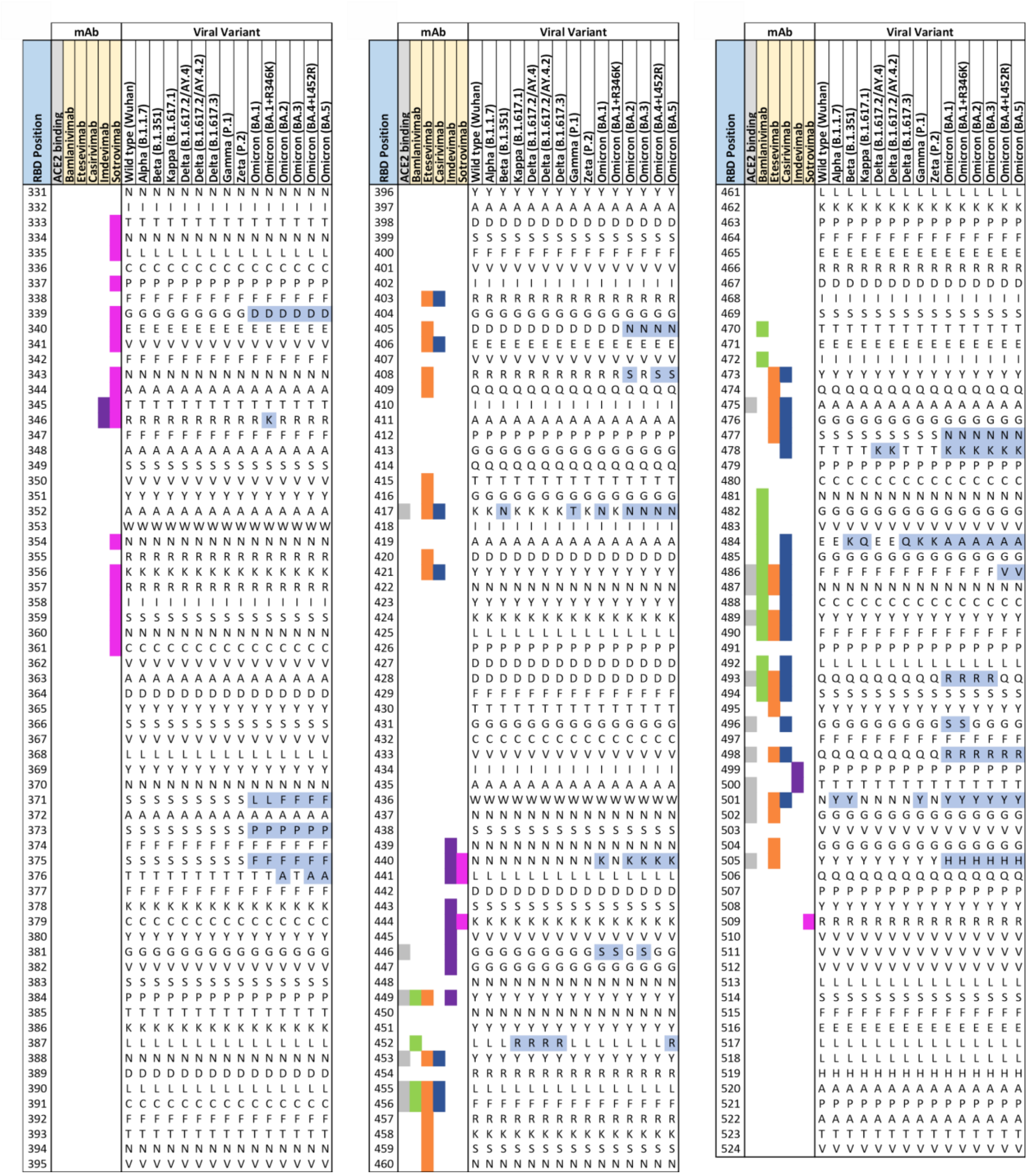

Supplemental Figure 2. Multiple-sequence alignment of Spike RBD protein sequences of different SARS-CoV-2 variants and binding sites for human ACE2 (grey), bamlanivimab (green), etesevimab (orange), casirivimab (blue), imdevimab (purple), and sotrovimab (magenta). Spike RBD sequences from Wuhan (NC_045512), Alpha (B.1.1.7: EPI_ISL_674612), Beta (B.1.351: EPI_ISL_940877), Kappa (B.1.617.1: EPI_ISL_1384866), Delta (B.1.617.2/AY.4: EPI_ISL_1758376, B.1.617.2/AY.4.2: OX014422; B.1.617.3: MZ359842), Gamma (P.1: EPI_ISL_2777382), Zeta (P.2: EPI_ISL_717936), and Omicron (BA.1: EPI_ISL_6795848, BA.1+R346K: EPI_ISL_8724963, BA.2: EPI_ISL_8135710, BA.3: OM508650, BA.4+L452R: EPI_ISL_11542550, BA.5: EPI_ISL_11542604) are displayed. Non-synonymous amino acid residues compared to the Wuhan reference are highlighted in blue. Adapted from ref. (1).

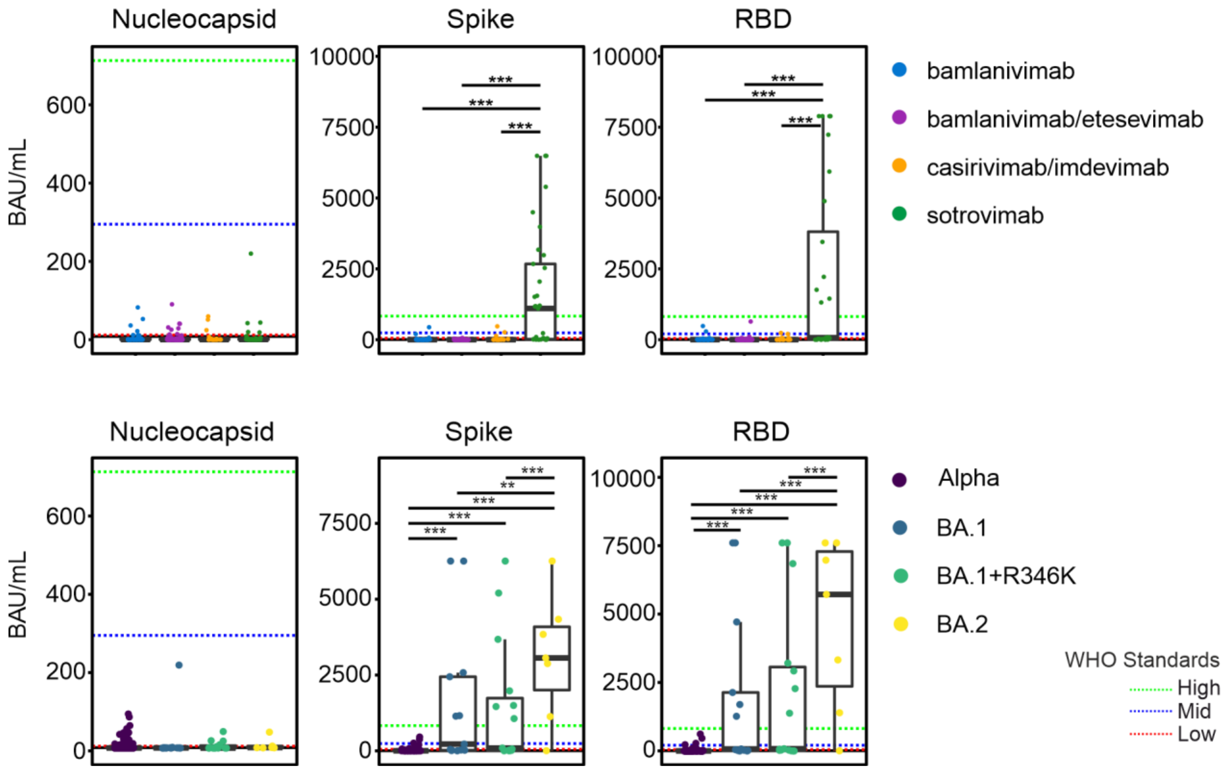

Supplemental Figure 3. Anti-N, anti-S, and anti-RBD serology titers of patients receiving mAb therapy at D0 stratified by therapy and variant. Red, green, and blue dotted lines indicate SARS-CoV-2 WHO reference standard values for low, medium, and high antibody titers, respectively. Longitudinal statistical comparisons were performed using Mann-Whitney followed by Bonferroni post-hoc correction. Box plots indicate median (middle line), 25th, 75th percentile (box), and 5th and 95th percentile (whiskers). All data points, including outliers, are displayed. **: $p < 0.01$. ***: $p < 0.001$. D0: sample collected prior to mAb infusion. BAU: Binding antibody units.

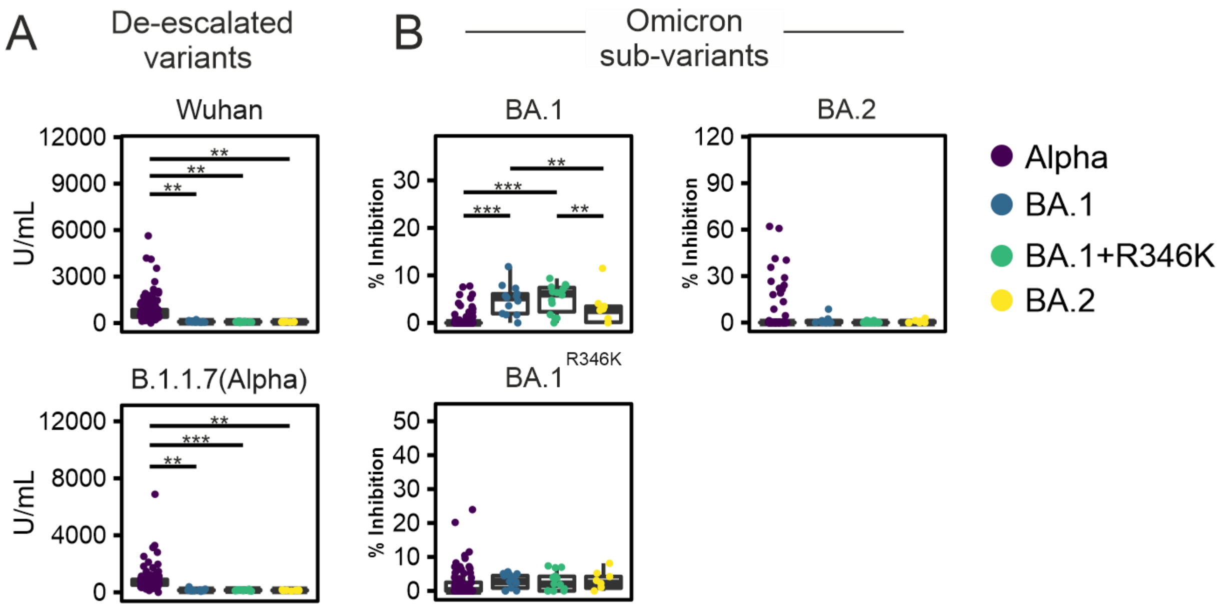

Supplemental Figure 4. Anti-S neutralization capacity of Alpha (B.1.1.7/Q4), BA.1, BA.1+R346K, and BA.2 was measured against (A) de-escalated variants, as well as (B) Omicron sub-variants at D2. Anti-S neutralizing antibody measurements against 5 different SARS-CoV-2 variants of concern in patients infected with Alpha, BA.1, BA.1+R346K, and BA.2 variants. Statistical assessments were performed using pairwise t-tests with Bonferroni post-hoc correction. Box plots indicate median (middle line), 25th, 75th percentile (box), and 5th and 95th percentile (whiskers). All data points, including outliers, are displayed. **: $p < 0.01$. ***: $p < 0.001$.

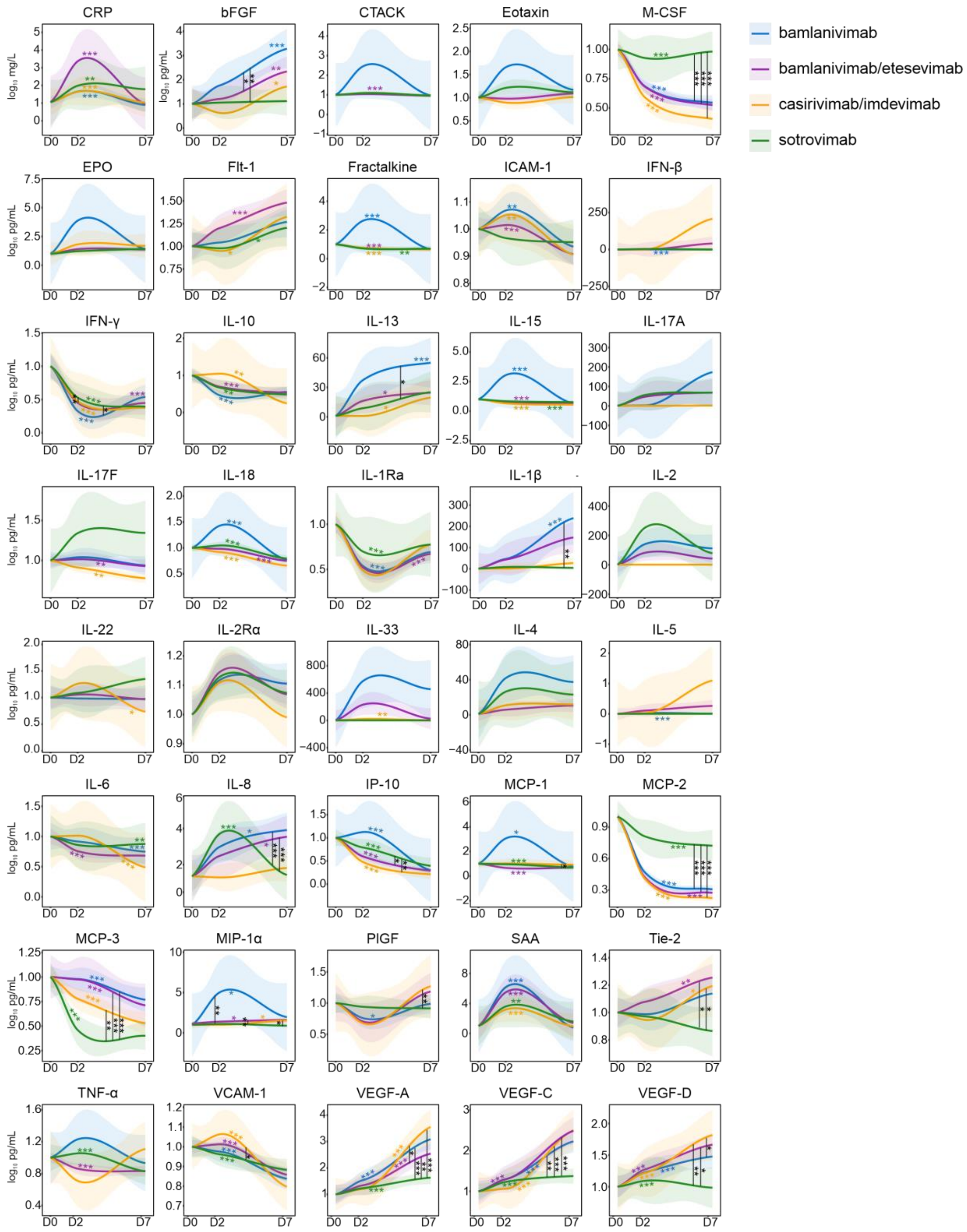

Supplemental Figure 5. Temporal evolution of circulating immune-related biomarkers (CIBs) in patients receiving bamlanivimab, bamlanivimab/etesevimab, casirivimab/imdevimab, or sotrovimab therapy. Time is represented as days after mAb therapy (D0, D2, and D7). Cross-sectional and longitudinal statistical comparisons were performed using Mann-Whitney followed by Bonferroni post-hoc correction. Lines represent smoothed conditional means for studied timepoints and shaded area display 95% confidence intervals for all measured timepoints. Colored asterisks in the graph refer to the significance of the slope from the 4 separate regression lines. Vertical lines with asterisks represent the significance of the pairwise comparison between the slopes in bamlanivimab, bamlanivimab/etesevimab, casirivimab/imdevimab, and sotrovimab therapy groups. D0: sample collected prior to mAb infusion. D2: 2 ± 1 days after mAb infusion. D7: 7 ± 2 days after mAb infusion. D28: 28 ± 4 days after mAb infusion. *: $p < 0.05$. **: $p < 0.01$. ***: $p < 0.001$.

A

Patient characteristics	Non mutation (n=17)	Mutation carriers (n=2)	P-value
**Sotrovimab therapy (Spike RBD amino acid substitution)**	**7 (41.2)**	**1 (K346R, L371S, P373S, F375S)**	**NA**
**Tixagevimab/cilgavimab therapy (Spike RBD amino acid substitution)**	**10 (58.8)**	**1 (R408S)**	**NA**
Male (%)	9 (52.9)	1 (50.0)	NS
Age (mean, IQR or range)*	66 (62-75)	62 (51-72)	NS
< 65 years	57 (52-64)	51 (51-51)	NS
≥ 65 years	76 (72-79)	72 (72-72)	NS
BMI (median, IQR or range)	28 (25-31)	22 (21-24)	NS
WHO progression severity scale – At enrolment (mean, IQR or range)*	2 (2-3)	3 (2-3)	NS
WHO progression severity scale – Worst (mean, IQR or range)*	2 (2-3)	3 (2-3)	NS
Days from symptoms onset to mAb infusion (mean, IQR or range)*	2 (1-3)	3 (2-3)	NS
sO2 % (mean, IQR or range)*	97 (96-98)	96 (94-97)	NS
Anti-SARS-CoV-2 vaccination (>2 weeks post-dose, ≥2 doses, %)	16 (94.1)	2 (100)	NS
**Ongoing COVID-related therapy** (prednisone, azithromycin, amoxicillin/clavulanate)	0 (0.0)	0 (0.0)	NS
**Immunocompromising condition (%)**	7 (41.2)	2 (100)	NS
Solid organ cancer (with ongoing therapy/ongoing stopped < 6 mo) (%)	1 (5.9)	0 (0.0)	NS
Hematologic cancer (with ongoing CHT/ongoing stopped < 6 mo) (%)	3 (17.6)	1 (50.0)	NS
Solid organ transplant recipients (%)	0 (0.0)	0 (0.0)	NS
Immunological diseases requiring immunosuppressive agents (%)	4 (23.5)	1 (50.0)	NS
**Other comorbidities**			
Diabetes (with or without damage) (%)	2 (11.8)	0 (0.0)	NS
Cardiovascular disease (ischemic/arrythmia/hypertension) (%)	9 (52.9)	0 (0.0)	NS
Chronic renal failure (with or without need of dialysis) (%)	0 (0.0)	0 (0.0)	NS
Chronic pulmonary diseases (%)	5 (29.4)	1 (50.0)	NS
Any neurological/vascular disease (%)	1 (5.9)	0 (0.0)	NS
**Viral variant**			
BA.1/Omicron (%)	3 (17.6)	0 (0.0)	NS
BA.1+R346K/Omicron (%)	1 (5.9)	1 (50.0)	
BA.2/Omicron (%)	7 (41.2)	0 (0.0)	
BA.4/Omicron (%)	1 (5.9)	0 (0.0)	
BA.5/Omicron (%)	2 (11.8)	0 (0.0)	
BE.1/Omicron (%)	1 (5.9)	1 (50.0)	

*: where n=2, ranges are displayed;NA: not applicable; NS: non-significant,

B

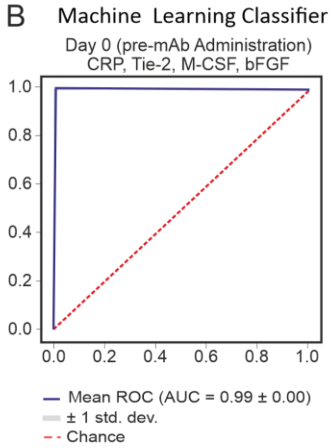

C

Regression classification

		Mutations No	Mutations Yes	Percentage Correct
Mutations	No	17	0	100
	Yes	0	2	100
Overall Percentage				100

Supplementary Figure 6. Out-of-sample performance of circulating immune-related biomarkers (CIBs) predicting *de novo* SARS-CoV-2 Spike RBD mutations in COVID-19 patients receiving mAb therapy. (A) Clinical characteristics of the enrolled patients for CIB validation. Statistical assessments of categorical and continuous variables were assessed across mAb therapy groups using chi-square tests of independence and analysis of variance (ANOVA), respectively. IQR: interquartile range. mo: months. **(B)** Utilizing random forest classification with SMOTE analysis based on a CIB panel comprising 4 biomarkers (CRP, Tie-2, M-CSF, and bFGF) before mAb treatment predicted de novo Spike RBD mutation development with AUROC of 0.99 within seven days of treatment. **(C)** Binomial logistic regression also predicted patients with or without de novo Spike RBD mutations with 100% accuracy.

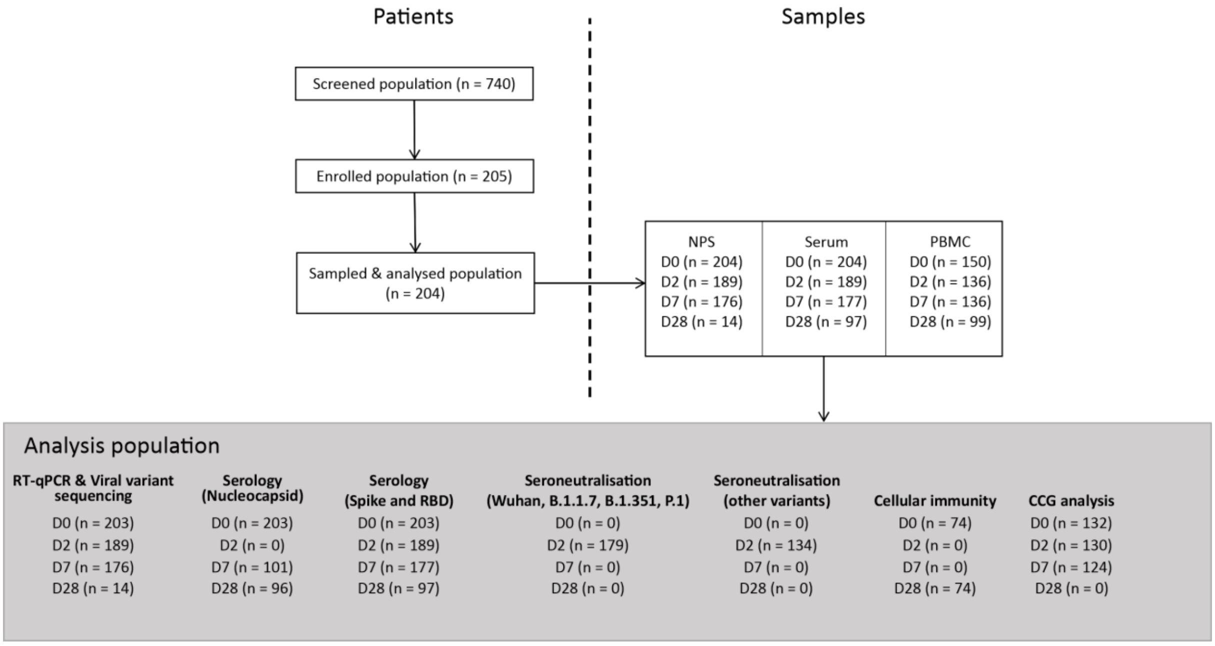

Supplemental Figure 7. Overview of patient and sample inclusion in the study. D0: sample collected prior to mAb infusion. D2: 2 ± 1 days after mAb infusion. D7: 7 ± 2 days after mAb infusion. D28: 28 ± 4 days after mAb infusion.

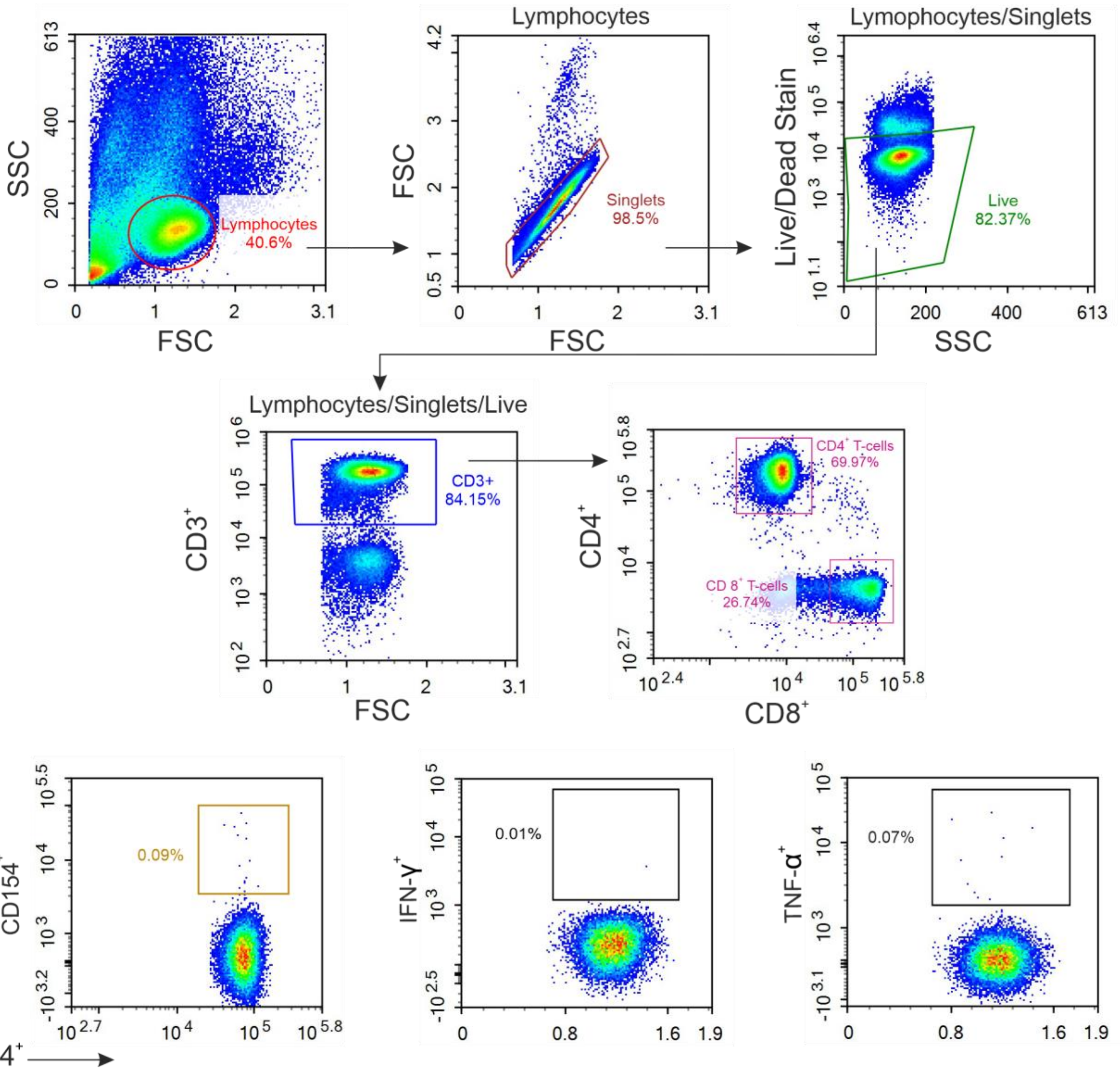

Supplemental Figure 8. Representative flow cytometry plots for analysis of activated CD4⁺ T helper (Th) cells and their expression of effector cytokines. Gating strategy after specific stimulation with either a SARS-CoV-2 Nucleocapsid or a complete Spike peptide pool. PBMCs were gated on lymphocytes. Singlets were gated with dead cells excluded. Live CD3⁺ T cells were identified. Within the CD4⁺ Th cell populations activated CD154⁺ Th cells were gated, and the expression of IFN-γ and TNF-α analyzed. PBMC: Peripheral blood mononuclear cells.

References

1. Arora P, Zhang L, Krüger N, Rocha C, Sidarovich A, Schulz S, et al. SARS-CoV-2 Omicron sublineages show comparable cell entry but differential neutralization by therapeutic antibodies. Cell host & microbe. 2022;30(8):1103-11.e6.
2. Savoldi A, Morra M, De Nardo P, Cattelan AM, Mirandola M, Manfrin V, et al. Clinical efficacy of different monoclonal antibody regimens among non-hospitalised patients with mild to moderate COVID-19 at high risk for disease progression: a prospective cohort study. European journal of clinical microbiology & infectious diseases : official publication of the European Society of Clinical Microbiology. 2022;41(7):1065-76.
3. Savoldi A, Morra M, Castelli A, Mirandola M, Berkell M, Smet M, et al. Clinical Impact of Monoclonal Antibodies in the Treatment of High-Risk Patients with SARS-CoV-2 Breakthrough Infections: The ORCHESTRA Prospective Cohort Study. Biomedicines. 2022;10(9):2063.
4. Shrestha LB, Foster C, Rawlinson W, Tedla N, and Bull RA. Evolution of the SARS-CoV-2 omicron variants BA.1 to BA.5: Implications for immune escape and transmission. Rev Med Virol. 2022:e2381.
5. Rockett R, Basile K, Maddocks S, Fong W, Agius JE, Johnson-Mackinnon J, et al. Resistance Mutations in SARS-CoV-2 Delta Variant after Sotrovimab Use. N Engl J Med. 2022;386(15):1477-9.
6. Birnie E, Biemond JJ, Appelman B, de Bree GJ, Jonges M, Welkers MRA, et al. Development of Resistance-Associated Mutations After Sotrovimab Administration in High-risk Individuals Infected With the SARS-CoV-2 Omicron Variant. JAMA. 2022.
7. Ragonnet-Cronin M, Nutalai R, Huo J, Dijokaite-Guraliuc A, Das R, Tuekprakhon A, et al. Genome-first detection of emerging resistance to novel therapeutic agents for SARS-CoV-2. bioRxiv : the preprint server for biology. 2022:2022.07.14.500063.
8. Weisblum Y, Schmidt F, Zhang F, DaSilva J, Poston D, Lorenzi JC, et al. Escape from neutralizing antibodies by SARS-CoV-2 spike protein variants. eLife. 2020;9.
9. Zhao Z, Zhou J, Tian M, Huang M, Liu S, Xie Y, et al. Omicron SARS-CoV-2 mutations stabilize spike up-RBD conformation and lead to a non-RBM-binding monoclonal antibody escape. Nature communications. 2022;13(1):4958.
10. Guigon A, Faure E, Lemaire C, Chopin MC, Tinez C, Assaf A, et al. Emergence of Q493R mutation in SARS-CoV-2 spike protein during bamlanivimab/etesevimab treatment and resistance to viral clearance. The Journal of infection. 2022;84(2):248-88.
11. Jary A, Marot S, Faycal A, Leon S, Sayon S, Zafilaza K, et al. Spike Gene Evolution and Immune Escape Mutations in Patients with Mild or Moderate Forms of COVID-19 and Treated with Monoclonal Antibodies Therapies. Viruses. 2022;14(2).
12. Jensen B, Luebke N, Feldt T, Keitel V, Brandenburger T, Kindgen-Milles D, et al. Emergence of the E484K mutation in SARS-COV-2-infected immunocompromised patients treated with bamlanivimab in Germany. Lancet Reg Health Eur. 2021;8:100164.
13. Simons LM, Ozer EA, Gambut S, Dean TJ, Zhang L, Bhimalli P, et al. De novo emergence of SARS-CoV-2 spike mutations in immunosuppressed patients. Transpl Infect Dis. 2022:e13914.
14. Choi B, Choudhary MC, Regan J, Sparks JA, Padera RF, Qiu X, et al. Persistence and Evolution of SARS-CoV-2 in an Immunocompromised Host. N Engl J Med. 2020;383(23):2291-3.
15. Scherer EM, Babiker A, Adelman MW, Allman B, Key A, Kleinhenz JM, et al. SARS-CoV-2 Evolution and Immune Escape in Immunocompromised Patients. N Engl J Med. 2022;386(25):2436-8.

Supplemental Acknowledgements

mAb ORCHESTRA working group: Basil Britto Xavier[2], Christine Lammens[2], Shahla Mahmoudi[1], Nour Chebly[2], Anna Gorska[3], Chiara Zanchi[3], Giulia Rosini[3], Rebecca Scardellato[3], Laura Rovigo[3], Francesco Luca[3], Lorenzo Tavernaro[3], Salvatore Hermes Dall'O[3], Chiara Konishi De Toffoli[3], Massimo Mirandola[3,5], Concetta Sciammarella[3], Riccardo Cecchetto[6], Davide Gibellini[6], Giuseppe Lippi[4], Matteo Gelati[4], Michela Conti[3]

[1]Molecular Pathology Group, Cell Biology & Histology, Faculty of Medicine and Health Sciences, University of Antwerp, Antwerp, Belgium
[2]Laboratory of Medical Microbiology, Vaccine & Infectious Disease Institute, University of Antwerp, Antwerp, Belgium
[3]Division of Infectious Diseases, Department of Diagnostics and Public Health, University of Verona, Verona, Italy
[4]Section of Clinical Biochemistry, University of Verona, Verona, Italy
[5]School of Health Sciences, University of Brighton, Brighton, United Kingdom
[6]Section of Microbiology, Department of Diagnostics and Public Health, University of Verona, Verona, Italy
